# Niche divergence at the intraspecific level in an endemic rare peony (*Paeonia rockii*): A phylogenetic, climatic and environmental survey

**DOI:** 10.3389/fpls.2022.978011

**Published:** 2022-11-01

**Authors:** Peng-Bin Dong, Ling-Juan Wang, Yun Jia, Zhong-Hu Li, Hong-Yan Wang, Feng-Xia Guo, Yuan Chen

**Affiliations:** ^1^ College of Agronomy, College of Life Science and Technology, State Key Laboratory of Arid land Crop Science, Gansu Agricultural University, Lanzhou, China; ^2^ College of Agriculture, Ningxia University, Yinchuan, China; ^3^ Xi’an Botanical Garden of Shaanxi Province (Institute of Botany of Shaanxi Province), Xi’an, China; ^4^ Key Laboratory of Resource Biology and Biotechnology in Western China (Ministry of Education) College of Life Sciences, Northwest University, Xi’an, China

**Keywords:** climate change, divergence time, migration prediction, *paeonia rockii*, phylogenetic relationship

## Abstract

Ecological factors have received increasing attention as drivers of speciation but also in the maintenance of postspeciation divergence. However, the relative significance of the responses of species to climate oscillations for driving niche divergence or conservatism in the evolution of many species that pass through diverse environments and limited geographical boundaries remains poorly understood. *Paeonia rockii* (one of the ancient species of *Paeonia*) comprising two subspecies called *Paeonia rockii* subsp. *rockii* and *Paeonia rockii* subsp. *taibaishanica* is an endemic, rare, and endangered medicinal plant in China. In this study, we integrated whole chloroplast genomes, and ecological factors to obtain insights into ecological speciation and species divergence in this endemic rare peony. RAxML analysis indicated that the topological trees recovered from three different data sets were identical, where *P*. *rockii* subsp. *rockii* and *P*. *rockii* subsp. *taibaishanica* clustered together, and molecular dating analyses suggested that the two subspecies diverged 0.83 million years ago. In addition, ecological niche modeling showed that the predicted suitable distribution areas for *P*. *rockii* subsp. *rockii* and *P*. *rockii* subsp. *taibaishanica* differed considerably, although the predicted core distribution areas were similar, where the population contracted in the last interglacial and expanded in the last glacial maximum. Under the emissions scenarios for the 2050s and 2070s, the suitable distribution areas were predicted to contract significantly, where the migration routes of the two subspecies tended to migrate toward high latitudes and elevations, thereby suggesting strong responses of the distributions of the two subspecies to climate change. These findings combined with the phylogeographic relationships provide comprehensive insights into niche variation and differentiation in this endemic rare peony, and they highlight the importance of geological and climatic changes for species divergence and changes in the population geographic patterns of rare and endangered medicinal plants in East Asia.

## Introduction

Global change, particularly global climate change, is among the most severe challenges facing humanity at present (Li, 1996). Since the industrial revolution, the concentration of greenhouse gases in the atmosphere has increased dramatically (Solomon et al., 2007) and caused global climate change to increase at an extremely rapid pace (Li and Chen, 2014). Global climate change is considered one of the greatest threats to natural ecosystems and it has significantly influenced species distributions and biodiversity at different spatial scales (Chen et al., 2011; Peng et al., 2021). As the problem of global climate change worsens, it is estimated that as many as 15–37% of species will be extinct by 2050 (Thomas et al., 2004). This situation is undoubtedly worse for those species with restricted geographic ranges, small population sizes, or high habitat specificity, and their populations are more likely to decrease and eventually become extinct (Jump and Penuelas, 2005; Daskalova et al., 2020). Therefore, determining the responses of rare and endangered species to climate changes in the past and future is helpful for understanding the historical causes of species formation and changes in geographical distributions, but also critical for assessing the vulnerability of biodiversity and guiding conservation efforts.

Under ongoing global climate change, ecological niche models (ENMs) have been widely used to assess the risk of invasive alien species, predict the potential distributions of species, determine the impacts of climate change on species, and to develop conservation strategies for endangered species (Renwick et al., 2018; Bates et al., 2020). ENMs have a statistically robust capacity for predicting the geographical distributions of species and they are important for research in ecology and biogeography (Johnson et al., 2019; Akiyama et al., 2020). In recent years, niche studies and observations have shown that climate change has modified the distribution patterns of species, where many species have migrated to high latitudes or high altitudes (Lehikoinen et al., 2019; Koebsch et al., 2020). Walther et al. (2007) studied the distribution dynamics of *Trachycarpus fortunei* and found that changes in winter temperatures and the growing season length caused this species to migrate northward, and they concluded that *T*. *fortunei* is an important bioindicator of global climate change (Walther et al., 2007). Chen et al. (2011) conducted a comprehensive survey of nearly 1,400 species, and found that global climate change is driving plants to migrate away from the Equator and toward the poles. Studies have shown that every 1°C increase in temperature shifts the tolerance limit of terrestrial species by 125 km toward the poles, or by 150 m in vertical elevation in mountainous areas (Lehikoinen and Virkkala, 2016). In addition to environmental factors such as temperature, changes in the distribution areas of species also depend on many factors such as the adaptability of the species itself, ability to migrate, obstacles in the migration process, and suitable distance for migration. For example, although the seeds of *Picea engelmannii* are small and can be spread by wind, it is estimated that they can only migrate 1–20 km/100 years when there are no obstacles (Li and Chen, 2014). Clearly, without anthropogenic assistance, the redistribution of many species may not be able to keep pace with climate change, and thus the structures and functions of ecosystems may be significantly altered. Many plant phylogeographic studies have been conducted in subtropical regions of China recently (Zhou et al., 2021), but the integrated responses of narrowly distributed populations, such as endemic, rare, and endangered species, to Quaternary climate change remain unclear in studies of complex phylogeographic structures. ENMs have become an indispensable tool for assessing the impact of climate change on species distribution patterns, inferring ice-age refuges for endangered species, and providing support for the management of endangered species, which will be of great theoretical and practical importance for the future conservation of biodiversity (Rhoné et al., 2020).

*Paeonia rockii* comprises two subspecies called *Paeonia rockii* subsp. *rockii* and *Paeonia rockii* subsp. *taibaishanica*, which belong to *Paeonia* section Moutan DC., Paeoniaceae. *P*. *rockii* is well known for its beauty, and it has important ornamental and economic value, as well as valuable medicinal properties (Hong et al., 2001). The root bark of *P*. *rockii* (Moutan Cortex, Mudanpi in Chinese) is one of the most widely used crude medications in China, with analgesic and anti-inflammatory properties (He and Dai, 2011; Zhang et al., 2014). In recent decades, the wild population resources of the two subspecies have rapidly decreased due to the effects of global climate change and human activities. These two subspecies of tree peony found in the Qinling Mountains and adjacent areas in central China have been listed as Class II protected plants in the China Red List of Biodiversity and the China Rare and Endangered Plants List (Wang, 2020). Previous studies by botanists and horticulturists have focused on the plant morphology, phytogeography, and phylogeny of members of Paeoniaceae (Hong, 2010; Zhou et al., 2021), but the phylogenetic relationships and molecular evolution of the subspecies *P*. *rockii* subsp. *rockii* and *P*. *rockii* subsp. *taibaishanica* remain unclear, and the study by Wu et al. (2021) based on the chloroplast genome found inconsistencies from morphological and taxonomic viewpoints (Wu et al., 2021). Moreover, niche differentiation is one of the most important factors for species differentiation (Clowers et al., 2015; Baniaga et al., 2020) but few studies have considered niche differentiation in the two *P*. *rockii* subspecies. Global climate change will lead to habitat loss and spatial isolation for rare and endangered plants, thereby increasing the risk of extinction (Gang et al., 2017; Lee et al., 2018). Therefore, it is necessary to investigate the historical changes in the distribution area of *P*. *rockii* under different climatic contexts in different periods and the future distribution and migration trends, thereby helping to elucidate the impact of climate change on the geographical distribution of this endangered plant and to provide a scientific basis for its protection.

The plastid genome is an organ that is independent of the nuclear genome (Wu et al., 2021). The plastid genome can be maternally inherited, the gene content and order are highly conserved, and it is characterized by slow molecular evolution and a low recombination rate, thereby making it an ideal material for species authentication and phylogenetic studies (Chumley et al., 2006; Drouin et al., 2008). Recently, whole plastid genome sequencing has emerged as a powerful method for resolving evolutionary relationships at multiple taxonomic levels, i.e., family and genus levels (Mader et al., 2020). In the present study, to clarify the phylogenetic relationships and population dynamics history of the two subspecies of *P*. *rockii*, we used newly sequenced and annotated plastid genomes sequences to determine the evolutionary relationships and evolution of Paeoniaceae species. In addition, we explored the potential impact of global climate change on the geographical distribution patterns of the two *P*. *rockii* subspecies. The specific objectives of this study were: (1) to investigate the phylogenetic relationships between these two subspecies and their evolutionary history; and (2) providing a theoretical basis for the study of ecological speciation and the evolution of East Asian flora.

## Materials and methods

### Plant samples and DNA sequencing

Samples of *P*. *rockii* subsp. *rockii* and *P*. *rockii* subsp. *taibaishanica* (fresh leaf tissues) were collected from Taibai Mountain Nature Reserve, Shaanxi Province, China, between July 3, 2021 and July 22, 2021 under permission from the government. All voucher specimens were deposited at Gansu Agricultural University Herbarium (GAUF), and detailed collection information is provided in **Table S1**. Total genomic DNA was extracted from leaf tissues dried with silica gel using the improved cetyl trimethyl ammonium bromide method (Doyle and Doyle, 1987). The quality of the DNA was assessed by agarose gel electrophoresis. We used an ultrasonicator to randomly fragment the extracted genomic DNA into 300–500 bp fragments. Library preparation was conducted using a NEBNext Ultra II DNA Library Prep Kit for Illumina (New England Biolabs, Ipswich, MA). Paired-end sequencing (2×150 bp) was conducted with the Illumina HiSeq 2500 platform.

### Assembly and annotation of plastid genomes

We used NGSQC Toolkit v2.3.3 to filter the raw data by removing low-quality sequences and contaminated sequences at the connector (Patel and Jain, 2012). The raw reads were assembled using SPAdes (Bankevich et al., 2012) and filtered with the GetOrganelle pipeline (Jin et al., 2018) based on plastid genome of the closely related species *P*. *rockii* subsp. *taibaishanica* (MW192444) as a reference. We used Geneious v8.0.2 (Kearse et al., 2012) and Bandage (Wick et al., 2015) for visualization to obtain a complete plastid genome sequence. The genome was automatically annotated with PGA (Qu et al., 2019) and DOGAM (Wyman et al., 2004). Finally, we used Circos to draw a physical map of the whole plastid genome and generate the plastid genome sequence information (Krzywinski et al., 2009). In addition, we downloaded the genomes of 14 members of Paeoniaceae and related species (*Corylopsis spicata*, *Disanthus cercidifolius* subsp. *longipes*, *Hamamelis mollis*, *Rhodoleia championii*, and *Sinowilsonia henryi*) from GenBank (**Table S2**).

### Phylogenetic analysis and divergence time estimation

In some cases, phylogenomics has been shown to be susceptible to systematic errors produced from a poor alignment of the data matrix and improper sequence evolution models (Jeffroy et al., 2006). In our analysis, several strategies have been applied to reduce the potential impact of systematic errors. Thus, three types of data sets were used to construct a phylogenetic tree comprising the whole plastome sequences (WP data set), protein-coding genes (PCG data set), and GBLOCKS v.0.91b (GBDN data set) was employed to remove ambiguously aligned sites in the whole plastid genome (Talavera and Castresana, 2007). For the WP data set, PCG data set, and GBDN data set, we employed the nucleotide sequences and aligned each data set using MAFFT v.7 (Katoh et al., 2005) with the default settings. MEGA X (Kumar et al., 2018) was then used to estimate the lengths of the aligned sequences, variant sites, parsimony signal sites, and singleton sites. Phylogenetic analyses were performed using the maximum likelihood method. For the WP data set, PCG data set, and GBDN data set, we used JModeltest v.2.1.1 (Darriba et al., 2012) to determine the best nucleotide substitution model (GTR+I+G) according to the Akaike’s information criterion. Finally, RAxML software was used to perform 1000 bootstrap repetitions for maximum likelihood analysis (Stamatakis, 2006).

In addition, to estimate the divergence times for members of Paeoniaceae, BEAST v.1.8 (Drummond et al., 2012) was used to estimate the node age and topological structure for Paeoniaceae plants based on the complete plastid genomes. BEAST analyses were conducted using the uncorrelated log-normal relaxed clock approach with a Yule tree prior and appropriate nucleotide substitution model (GTR + G + I). The divergence times were estimated by Markov chain Monte Carlo analysis for 50,000,000 generations, with sampling every 1,000 generations. The stationarity of the chains and convergence of the two runs was monitored by Tracer v. 1.5, determining whether the effective sample size (ESS) of all parameters was larger than 200 as recommended. Chronograms with nodal heights and 95% highest posterior density intervals were generated with TreeAnotator v. 1.7.5 (the first 5000 trees were discarded as a burnin) and displayed using FigTree v. 1.0. Two fossil points in Saxifragales and Hamamelidaceae were used in molecular clock analysis to estimate the divergence times. The first calibration point was the age of Saxifragales (lognormal distribution with mean = 1.5, standard deviation (sd) = 0.5, and offset = 95.25 Ma; Hermsen et al., 2003). The second calibration point was set with all Hamamelidaceae species using a normal distribution with mean = 84 Ma and sd = 1 (Zhou et al., 2001).

### Geographic distributions of species

We obtained the distributions of *P*. *rockii* subsp. *rockii* and *P*. *rockii* subsp. *taibaishanica* from the Chinese Virtual Herbarium (http://www.cvh.org.cn/), Global Biodiversity Information Facility database (http://www.gbif.org), Flora of China, the flora for various places, resource survey reports, previous studies, and field survey records obtained by our group. Each record was checked to ensure the availability of latitude and longitude information, and geographic information, and specimens that lacked latitude and longitude or elevation records were reconstructed and checked with Google Earth according to specific small geographical records. In total, 70 specimen record points for the species were finally screened, with 44 for *P*. *rockii* subsp. *rockii* and 26 for *P*. *rockii* subsp. *taibaishanica* (**Figure S1**).

### Climate data and environmental data screening

The WorldClim global climate database (https://www.worldclim.org) was used to obtain climate data for five historical period comprising 19 bioclimatic layers for the LIG (120,000–140,000 years), last glacial maximum (LGM, 22,000 years), current period (1970–2000), and future periods (2050s and 2070s). The spatial resolution used for the climate data was 2.5 arc-min (Hijmans et al., 2005). The LGM data were obtained from the CCSM and MIROC climate models. To simulate the future distribution of two subspecies, we used Representative Concentration Pathways (RCPs) and Shared Socio-economic Pathways (SSPs) emissions scenarios. The outputs of simulated precipitation, and temperatures from three high-resolution General Circulation Models were used in this study (MIROC5, BCC-CSM2-MR, and BCC-CSM1.1). BCC-CSM1.1 and MIROC5 climate change modeling data under the Representative Concentration Pathways (RCPs) 2.6, 4.5 and 6.0 proposed by the Intergovernmental Panel on Climate Change were used for the years 2050 and 2070 (Zhang et al., 2020). Studies have the high RCP8.5 pathway may overestimate the future supply of fossil fuels (Wang et al., 2017). Indeed, a previous report on the use of coal in the future that 90% of fossil fuels will be used 2070 (Rutledge, 2011), so we selected RCP6.0 and assumed that greenhouse gases will peak in 2080 (Ford et al., 2012). In addition, three different scenarios SSP126, SSP245 and SSP370 under BCC-CSM2-MR model of the Coupled Model Intercomparison Project Phase 6 (CMIP6) were selected as future (2050s and 2070s) climate models.

Strong correlations between environmental variables will lead to model overfitting and affect their contributions to MaxEnt model evaluation. To avoid overfitting of the results due to high covariance of the environmental variables, the Pearson correlation coefficients between the environmental variables were calculated using SPSS Statiatics software (Herrando-Moraira et al., 2019). If the correlation coefficient value of the two environmental factors is greater than |0.8|, only the variable with the higher contribution is retained and used in the MaxEnt model (Garza et al., 2020). Ultimately, nine climate variables (bio3, bio4, bio8, bio11, bio12, bio15, bio17, bio18, and bio19) were selected to build the model (**Table 1**).

**Table 1:** Description of environmental variables used in MaxEnt.

Type	Variable	Code
climate	Isothermality	bio3
	Temperature seasonality (standard deviation)	bio4
	Mean temperature of wettest quarter	bio8
	Mean temperature of coldest quarter	bio11
	Annual precipitation	bio12
	Precipitation seasonality (coefficient of variation)	bio15
	Precipitation of driest quarter	bio17
	Precipitation of the warmest quarter	bio18
	Precipitation of the coldest quarter	bio19

### Differences in bioclimatic layers

In order to assess the adaptive differences between *P*. *rockii* subsp. *rockii* and *P*. *rockii* subsp. *taibaishanica*, we used the nonparametric Kruskal–Wallis test to analyze the differences among the nine bioclimatic layers for the species, and plotted the kernel density and a box line diagram. R3.6.0 was used to perform nonparametric tests and to prepare nuclear density maps.

### ENM analysis

Based on the data collected from the specimen points and environmental variables, we assessed the niche conservativeness of *P*. *rockii* subsp. *rockii* and *P*. *rockii* subsp. *taibaishanica* by conducting niche equivalence and similarity tests. Niche conservatism or differences were evaluated between *P*. *rockii* subsp. *rockii* and *P*. *rockii* subsp. *taibaishanica* based on the geographic space (G-space) and environmental space (E-space).

The distribution records of *P*. *rockii* subsp. *rockii* and *P*. *rockii* subsp. *taibaishanica* species and bioclimatic data were imported into the MaxEnt 3.4.1 program for a modeling analysis (Phillips et al., 2017). To improve the accuracy of the predictions, we randomly selected 75% of the distribution points as the training data and the remaining 25% as the test data, the model was trained for 100 repetition and, the output data format set to Logistic and other values as default (Moreno et al., 2011). The accuracy of the MaxEnt model is evaluated by the area under the curve (AUC) value of the Receiver Operator Characteristic (ROC) (Hebbar et al., 2022). AUC >0.7 is generally considered to be a good model performance (Rebelo et al., 2010). Jackknifing was used to evaluate the contributions of the nine main bioclimatic layers that could affect the current distributions of *P*. *rockii* subsp. *rockii* and *P*. *rockii* subsp. *taibaishanica*. Finally, according to the actual geographical distribution of two subspecies in China, and with reference to the assessment of the existence probability in the IPCC report, the natural segment method was used to classify suitable areas into the following four categories: non-suitability (*p* < 0.049), low suitability (0.049≤*p* < 0.196), moderately suitability (0.196 ≤ *p* < 0.441) and high suitability (0.441≤*p* < 1), which are indicated by different colours.

E-space niche model analysis was conducted to assess the similarity of the climatic niche spaces occupied by each species according to the method described by Broennimann et al. (2011). Following the approach initially proposed by Broennimann et al. (2011), principal component analysis (PCA) was used to translate the occurrence and climate data into environmental axes (PCA-env). The R package ECOSPAT was used to further evaluate the ENMs for each species (Cola et al., 2017). The advantages of this approach are that it can explain the spatial resolution biases, optimize the utilization of geographic and environmental space, and correct the observed occurrence density according to the availability of environmental space (Broennimann et al., 2011). We explored the similarity of the environmental niches of the *P*. *rockii* subsp. *rockii* and *P*. *rockii* subsp. *taibaishanica* using Schoener’s D niche overlap metric, which ranges from 0 (no overlap) to 1 (complete overlap) (Warren et al., 2008). To quantify changes in niches, we calculated three indices of niche dynamics (unfilling, stability and expansion). Niche stability is the niche overlap between species, niche expansion describes new climate conditions occupied by the species in one of its ranges, niche unflling classically refers to that climate available in invaded ranges but not yet occupied (Herrando-Moraira et al., 2019), and thus we conducted quantitative analyses using these three ecological niche dynamic indicators.

### Centroid displacement in suitable distribution areas

In order to obtain an overall understanding of the changes in the distributions, the center point of a suitable distribution area was calculated, and a vector diagram was drawn to illustrate the direction and distance of the changes in the center points for the two subspecies under different climate conditions. The SDM_Toolbox_v2.4 package was used to calculate the positions of the centroids of the suitable species areas, and the changes in the centroid positions under different climate change scenarios in the LIG, LGM, current period, 2050s, and 2070s were compared to calculate the distance of centroid migration (Chen et al., 2020). First, the predicted potentially suitable areas for the species obtained by the simulation were converted into a binary vector, i.e., the species suitability probability *p* °C 0.5 was set as the total suitable area and *p* < 0.5 as the non-suitable area. The spatial analysis tool was the used to calculate the position coordinates of the centroids of the suitable species areas. Finally, we tracked the centroids with different SDMs to examine the centroids for *P*. *rockii* subsp. *rockii* and *P*. *rockii* subsp. *taibaishanica* in different periods and under different climatic conditions to evaluate the migration distances of the suitable areas for the two subspecies in latitude and longitude coordinates.

## Results

### Genomic features of plastomes and data sets

**Figure 1:**
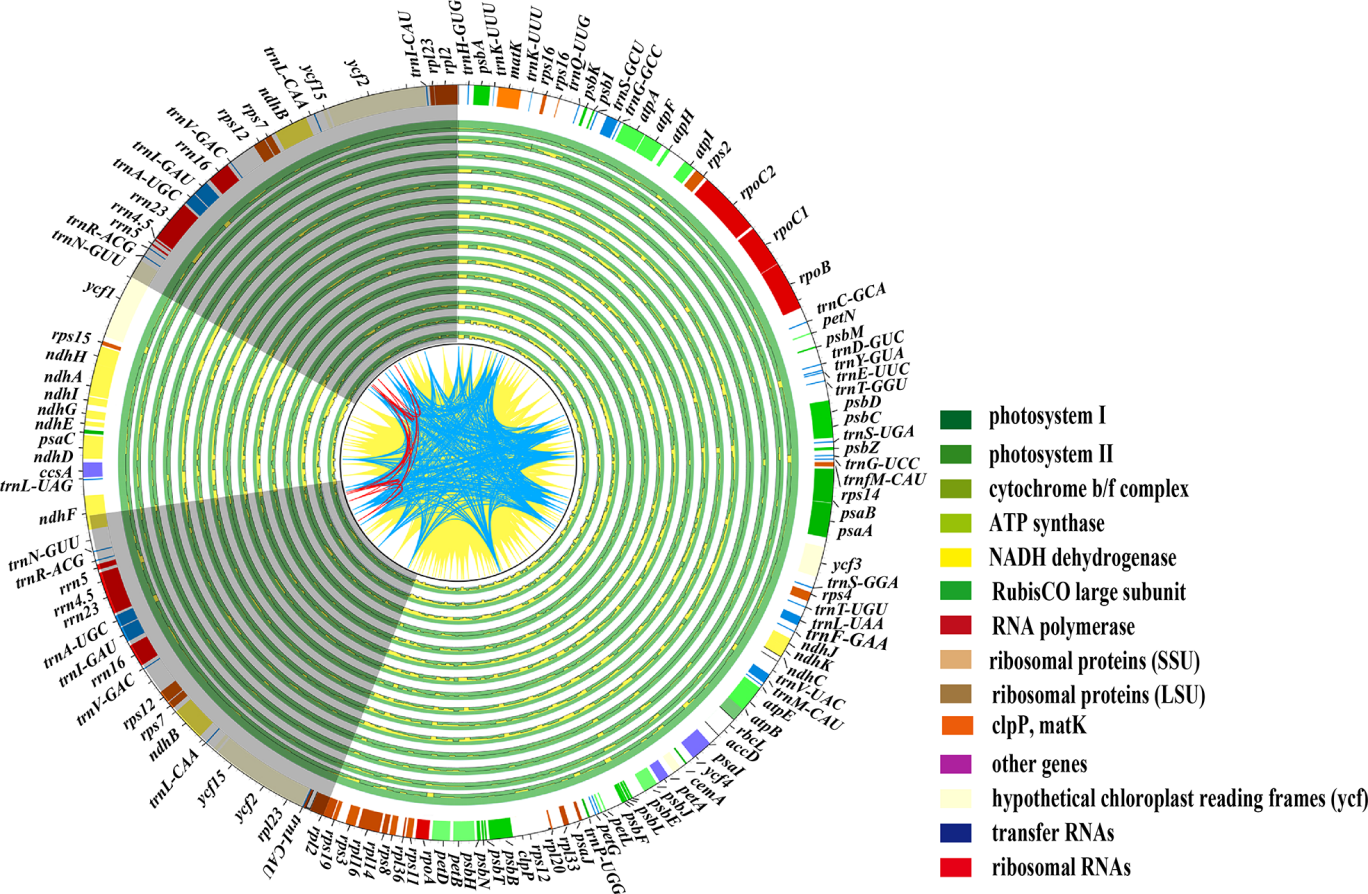
shows plastome variations within the Paeoniaceae family, with the plastid genome of *P*. *rockii* subsp. *taibaishanica* (MW192444) as a reference. The two IR regions (IRa and IRb) are represented with a grey backdrop in the quadripartite structure of the plastomes, but the large small single-copy regions (LSC and SSC) are displayed with a blank background. The lines from CDS to CDS are filled with yellow ridges, whereas the lines from tRNA to tRNA are shown with blue ridges, and other red lines are from rRNA to rRNA. The identical sites are filled with green ridges whereas the variations are shown with yellow ridges.

The plastid genomes of 16 *Paeonia* species accessions had exhibited a typical quadripartite structure (**Figure 1**), where the large single-copy lengths ranged from 84,242 bp (*P*. *qiui*) to 85,030 bp (*P*. *rockii* subsp. *taibaishanica*), small single-copy lengths ranged from 16,679 bp (*P*. *brownii*) to 17,077 bp (*P*. *ostii*), and inverted repeat region lengths ranged from 25,639 bp (*P*. *ludlowii*) to 25,745 bp (*P*. *suffruticosa*) (**Table S3**). In addition, the chloroplast genome of two subspecies shows a typical tetrad structure with a total length ranging from 152,840 bp (*P*. *rockii* subsp. *rockii*) to 153,368 bp (*P*. *rockii* subsp. *taibaishanica*). Furthermore, 123 functional genes were identified in two subspecies plastid genomes, i.e., 83 protein-coding genes, 37 tRNA genes, and eight ribosomal RNA genes (**Figure S2**). The WP data set had an aligned length of 168,986 bp and 12,418 parsimony-informative character (PICs) sites were detected across Saxifragales (**Table 2**). Removing ambiguous sites (GBDN) reduced this number to 140,577 total aligned nucleotides, yielding 10,038 PICs (**Table 2**). The PCG data set had an aligned length of 83,772 nucleotides with 4,759 PICs (**Table 2**).

**Table 2:** Data characteristics, selected model in each data set.

Data set	Numberof sites	Variable sites (%)	Parsimony-informativesites (%)	Singleton sites (%)	Best fit model
WP	168,986	19,970 (11.82)	12,418 (7.35)	7,405 (4.38)	GTR + I + G
PCG	837,72	8,159 (9.74)	4,759 (5.68)	3,380 (4.03)	GTR + I + G
GBDN	140,577	15,030 (10.69)	10,038 (7.14)	4,992 (3.55)	GTR + I + G

### Phylogenetic relationships and divergence time estimation

**Figure 2:**
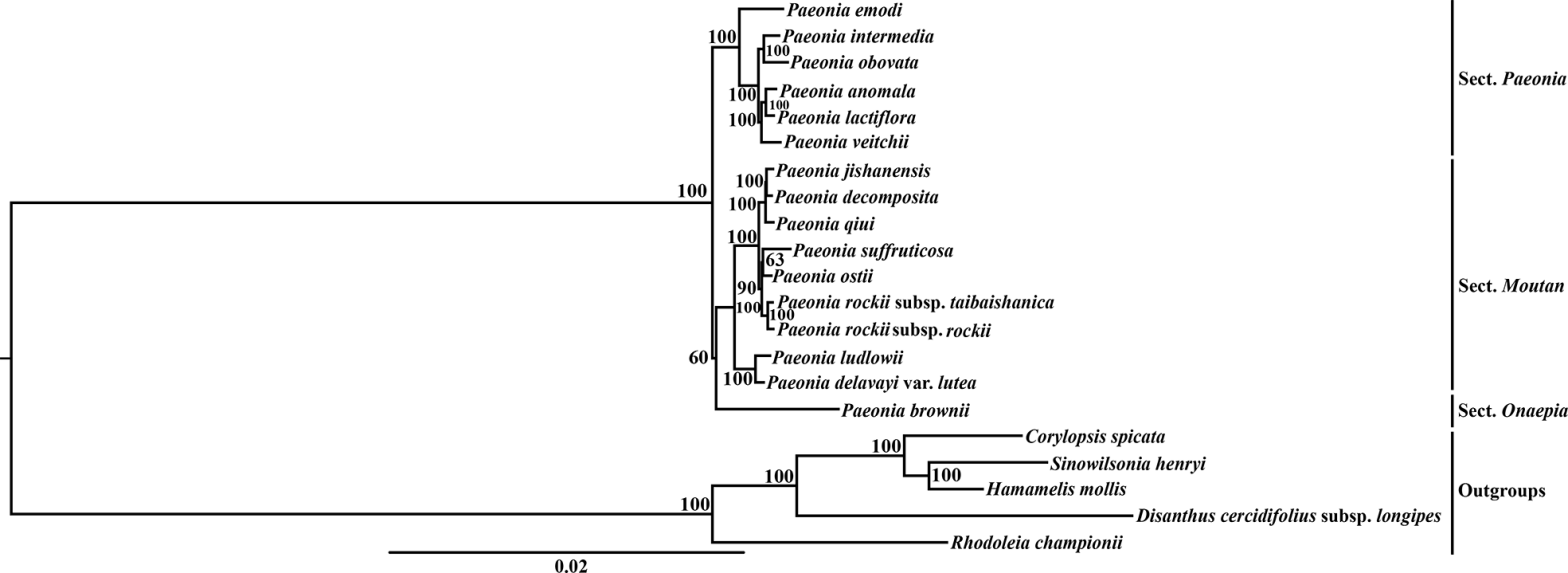
The maximum likelihood (ML) tree of Paeoniaceae inferred from the whole plastome data set. Numbers at nodes correspond to ML bootstrap percentages (100 replicates, only values greater than 50% are shown).

Different matrices were prepared based on the WP, GBDN, and PCG data sets, and three independent phylogenetic trees were reconstructed for Paeoniaceae. Phylogenetic analysis showed that the same topological tree was recovered from each data set and all nodes had high bootstrap values (**Figures 2**, **S3**, **S4**). All analyses completely resolved the phylogenetic relationships among the major clades and within the species in Paeoniaceae. The trees were largely congruent with the morphology and classification, thereby suggesting that *Paeonia* species could be divided into three large sub-clades corresponding to the three sections of *Paeonia*, *Moutan*, and *Onaepia*. The species in the section *Paeonia* clustered in one clade, which was further divided into different subclades. *P*. *emodi* was located at the base. *P*. *anomala*, *P*. *lactifora*, and *P*. *veitchii* clustered into a subclade and formed a sister relationship with the subclade containing *P*. *intermedia* and *P*. *obovata*. In section *Moutan*, species in subsection *Vaginatae* and subsection *Delavayanae* clustered in different branches. *P*. *jishanensis*, *P*. *decomposita*, *P*. *qiui*, *P*. *suffruticosa*, *P*. *ostii*, *P*. *rockii* subsp. *taibaishanica*, and *P*. *rockii* subsp. *rockii* clustered together and formed a sister relationship with the subclade containing *P*. *ludlowii* and *P*. *delavayi* var. *lutea.* We only sampled one species (*P*. *brownii*) from section *Onaepia* and it was placed at the basal position of the phylogenetic tree.

**Figure 3:**
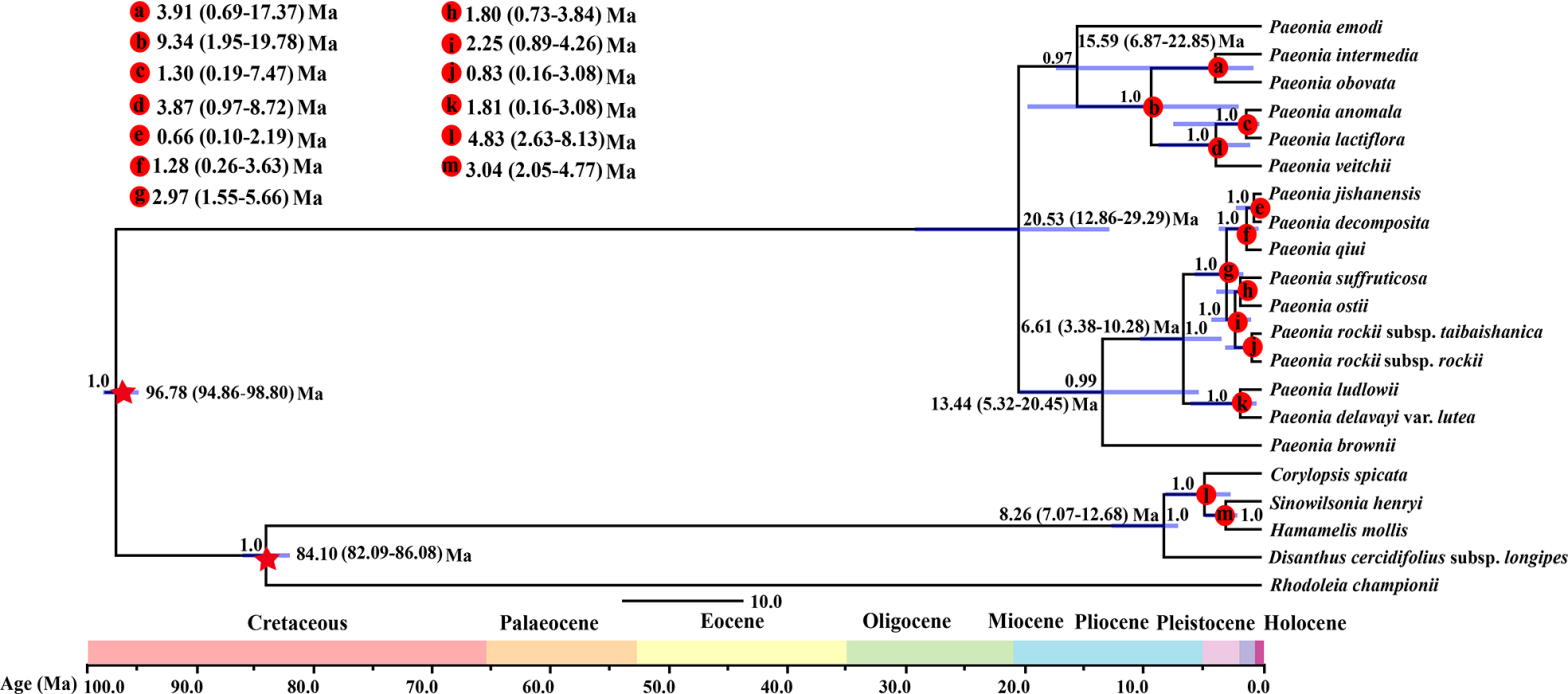
Chronogram for the Paeoniaceae species obtained using BEAST based on the plastid genome. The 95% highest posterior density (HPD) credibility intervals for node ages are labeled above the line.

Based on the whole plastome sequences, the fossil-corrected molecular clock model showed that the molecular phylogenetic tree crown age of Paeaceae was 20.53 Ma (95% highest posterior density (HPD): 12.86–29.92 Ma) (**Figure 3**). Section *Moutan* and section *Onaepia* formed obvious sister branches with a divergence time about 13.44 Ma (95% HPD: 5.32–20.45 Ma). Section *Moutan* contained strongly supported distinct clades, where *P*. *rockii* subsp. *rockii* and *P*. *rockii* subsp. *taibaishanica* diverged at 0.83 Ma (95% HPD: 0.16–3.08 Ma) in the Late Pleistocene.

### Climatic variables

**Figure 4:**
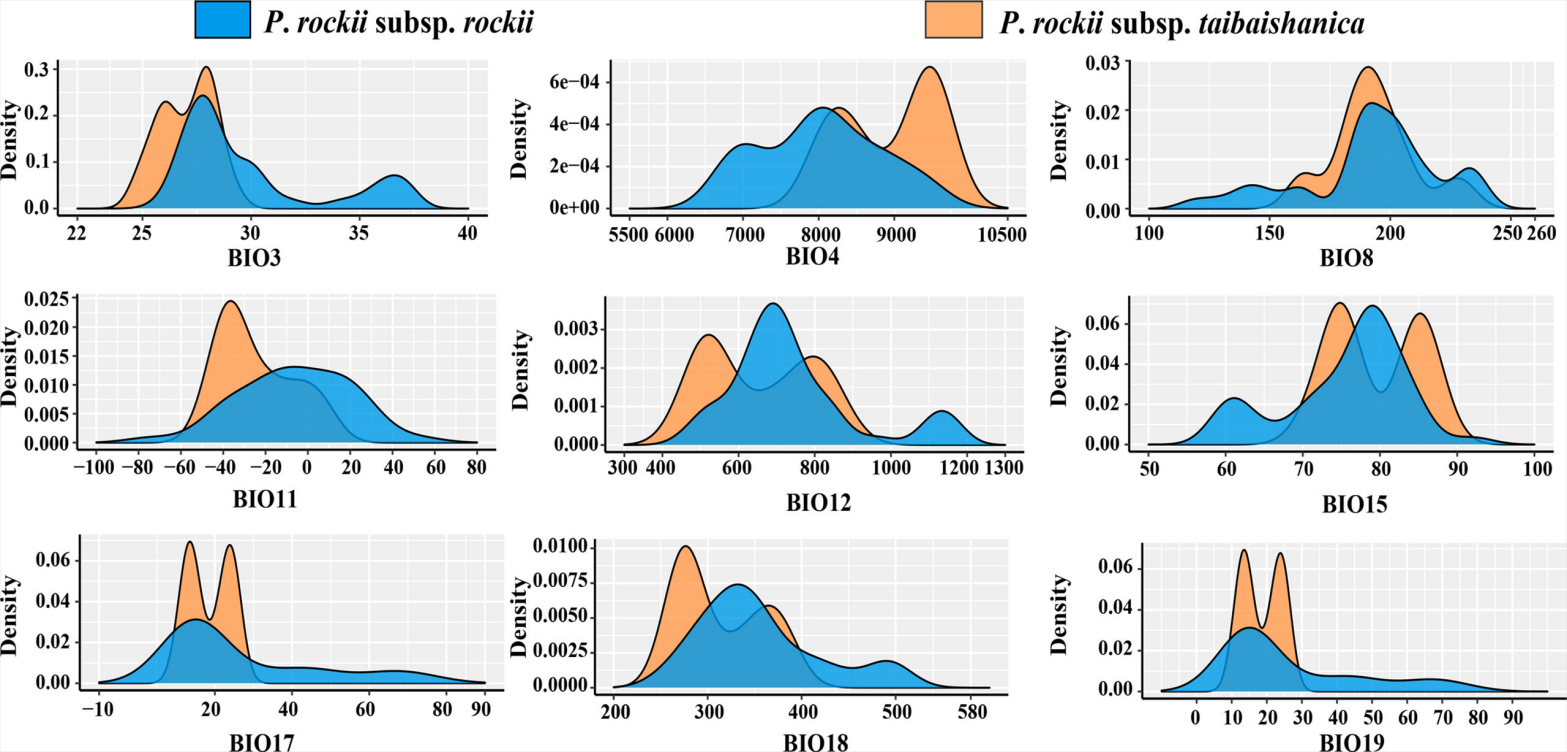
Kernel density plots of nine climatic variables for two subspecies of *P*. *rockii*.

Analysis of the environmental climatic variables using the Kruskal–Wallis test to compare the climatic niches of the two subspecies found two variables with highly significant differences (*p* < 0.01; bio3 and bio4), two variables with significant differences (*p* < 0.05; bio11 and bio18), and the other five variables were not statistically different (*p* > 0.05; bio8, bio12, bio15, bio17, and bio18) (**Figures 4**, **S5**). Jackknifing analysis was used to determine the contributions of the nine climate factor models in order to identify the main environmental factors that affected the current distributions of the two subspecies. As shown in **Figure S6**, the contributions of bio11, bio15, and bio19 to the current potential distribution of *P*. *rockii* subsp. *rockii* were obtained as 51%, 15.6%, and 14.4%, respectively, and with the cumulative contribution exceeded 80%. The contributions of bio11, bio17, and bio3 to the current potential distribution of *P*. *rockii* subsp. *taibaishanica* were obtained as 65.1%, 13.8%, and 11.8%, respectively, and the cumulative contribution was 90.7%.

### ENMs

**Figure 5:**
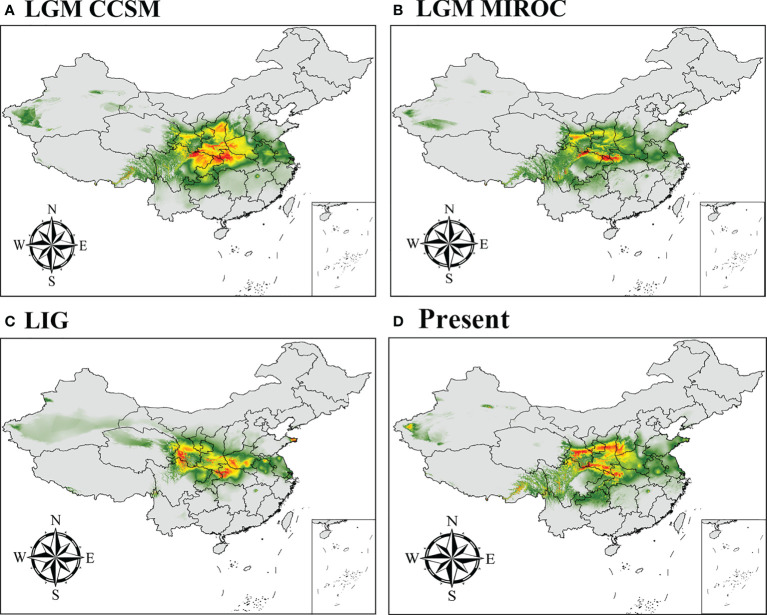
Potential distribution for *P*. *rockii* subsp. *rockii* during different periods predicted by the MaxEnt model based on optimized parameters. The potential distribution of *P*. *rockii* subsp. *rockii* was divided into four grades by the natural breaks method. Gray, green, yellow, and red areas represent not suitable, marginally suitable, moderately suitable, and highly suitable areas, respectively.

We predicted the current, LGM, and LIG distributions for *P*. *rockii* subsp. *rockii* and *P*. *rockii* subsp. *taibaishanica*, as shown in **Figures 5**, **S7**. Using the natural breaks method, the potential distributions of the two subspecies were divided into four grades (not suitable, marginally suitable, moderately suitable, and highly suitable areas). According to the ENM results, the predicted suitable distribution areas differed considerably for the two subspecies, but the predicted core distribution areas were similar (**Figures 5**, **S7**). During the LIG, *P*. *rockii* subsp. *rockii* would have occurred mainly in southern Gansu, northern Sichuan, southern Shaanxi, southwestern Henan, and northwestern Hubei in China (**Figure 5C**), where the area of the high suitability area was 2.61×10^4^ km^2^ smaller, the area of the medium suitability area was 11.83×10^4^ km^2^ smaller, and the area of the low suitability area was 29.67×10^4^ km^2^ larger (**Table 3**). *P*. *rockii* subsp. *taibaishanica* was mainly distributed in northern Xinjiang, Ningxia, northern Sichuan, the Ziwu Ridge in northern Shaanxi, and southeastern Shaanxi (**Figure S7C**), where the area of the high suitability area was 6.92×10^4^ km^2^ smaller, the area of the medium suitability area was 16.48×10^4^ km^2^ smaller, and the area of the low suitability area was 7.02×10^4^ km^2^ larger (**Table 3**). During the LGM, the distributions of the two subspecies expanded to the widest geographic distributions and largest areas. The core area for *P*. *rockii* subsp. *rockii* was mainly concentrated in the Ziwu Ridge in northern Shaanxi, Qinling Mountains in southern Shaanxi, western Sichuan, western Henan, northern Chongqing, and northern Hubei (**Figure 5A**). The core distribution habitat for *P*. *rockii* subsp. *taibaishanica* was focused in eastern Xinjiang, southern Shaanxi, and northeastern Sichuan (**Figure S7A**).

**Table 3:** The potential distribution area of the two *Paeonia* species in different periods.

Species	Period	Area of each suitable region (× 10^4^ Km^2^)			
		Marginally suitable region	Moderately suitable region	Highly suitable region	Total sutable region
*Paeonia rockii* subsp.* rockii*	LGM (CCSM)	97.13 (+1.71)	61.80 (+10.17)	55.61 (+29.82)	214.54 (-41.7)
	LGM (MIROC)	98.80 (+3.38)	60.77 (+9.14)	24.34 (-1.45)	183.91 (+11.07)
	LIG	65.75 (-29.67)	39.80 (-11.83)	23.18 (-2.61)	128.73 (-44.11)
	Current	95.42 (0.00)	51.63 (0.00)	25.79 (0.00)	172.84 (0.00)
*Paeonia rockii* subsp. *taibaishanica*	LGM (CCSM)	162.45 (+92.62)	98.35 (+55.52)	31.75 (+7.9)	292.55 (+156.04)
	LGM (MIROC)	104.17 (+34.34)	88.78 (+45.95)	27.74 (+7.9)	220.69 (+84.18)
	LIG	76.85 (+7.02)	26.35 (-16.48)	16.93 (-6.92)	120.13 (-16.38)
	Current	69.83 (0.00)	42.83 (0.00)	23.85 (0.00)	136.51 (0.00)

**Figure 6:**
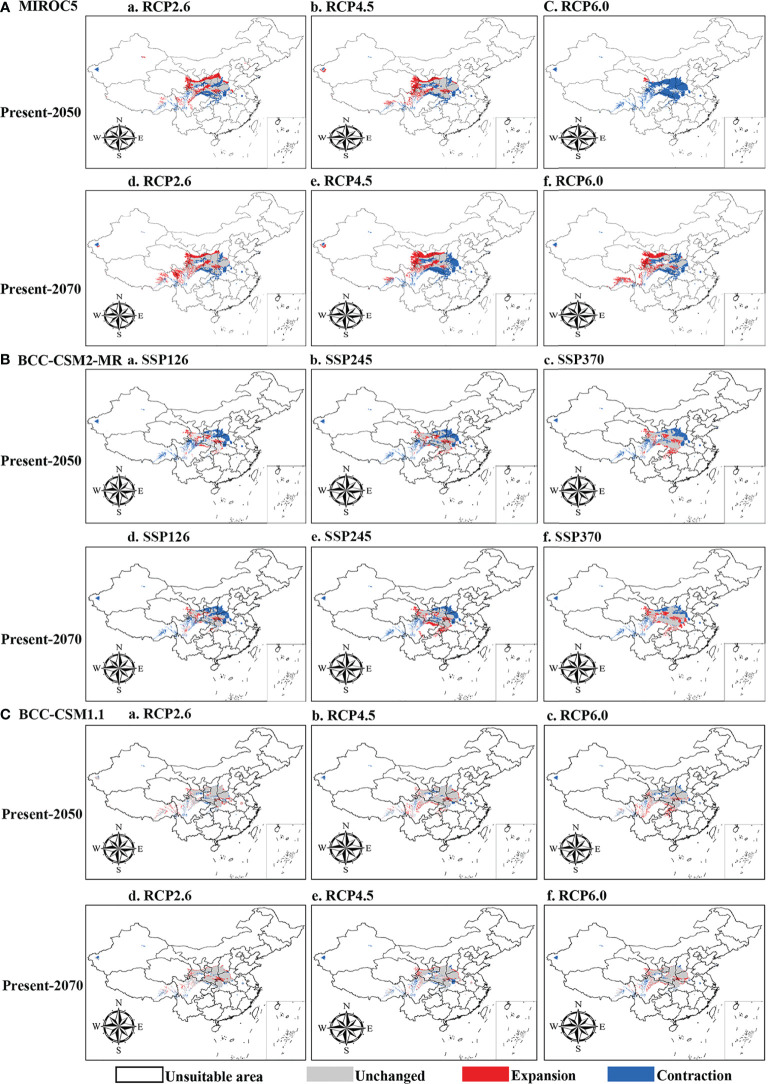
Spatial changes of *P*. *rockii* subsp. *rockii* in China under emission scenarios of the 2050s and 2070s. White, gray, red and blue areas represent not suitable, unchanged suitable, expansion suitable, and contraction suitable areas, respectively.

The current distribution areas of the two subspecies were captured well by the niche model. The results indicate that the current predicted core areas and current distribution sites were generally consistent with the highly suitable areas in the Ziwu Ridge in northern Shaanxi, Qinling Mountains in southern Shaanxi, Southern Gansu, Ningxia, northern Chongqing, and northern Hubei (**Figures 5D**, **S7D**). The three representative concentration pathways (RCP2.6, RCP4.5, and RCP6.0) were then modeled for two future time periods (2050 and 2070) (**Table 4**). Under future climatic conditions, three high-resolution General circulation models predict that some suitable areas for the two subspecies are expected to expand, but the total suitable area is predicted to contract (**Figures 6**, **S8**, and **Tables 5**, **S4**). In 2050, compared with the current potential distribution area, *P*. *rockii* subsp. *rockii* will have a reduced range in western Xinjiang, southern Gansu, southern Shaanxi, and southwestern Shanxi in China, and it will gradually expand into the margins of the Sichuan Basin, Henan, eastern Qinghai and eastern Tibet (**Figure 6**). *P*. *rockii* subsp. *taibaishanica* is predicted to undergo large-scale expansion in Xinjiang, Shaanxi and southwestern Shanxi (**Figure S8**). Compared with the present distributions, the migrations and expansions determined the two subspecies in 2070 were consistent with the results for 2050.

**Table 4:** Three emission scenarios using in this study.

Emission	Description
RCP2.6	The radiative forcing reached its peak before 2100 and decreased to 2.6 W/m^2^ by 2100. The peak CO_2_ equivalent concentration was about 490 mL/m^3^.
RCP4.5	The radiative forcing stabilized at 4.5 W/m^2^, and the CO_2_ equivalent concentration stabilized at about 600 mL/m^3^ after 2100.
RCP6.0	The radiative forcing stabilized at 6.0 W/m^2^, and the CO_2_ equivalent concentration stabilized at about 850 mL/m^3^ after 2100.

**Table 5:** The potential distribution area of the two *Paeonia* species in the 2050s and 2070s.

Species	Period	Area of each suitable region (× 10^4^ Km^2^)			
		Unsuitable region	Unchanged region	Expansion region	Contractionregion
*Paeonia rockii* subsp.* rockii*	Current **vs** RCP2.6-2050s	891.75	51.04	8.57	8.64
	Current **vs** RCP4.5-2050s	887.18	51.49	8.12	13.21
	Current **vs** RCP6.0-2050s	888.99	48.77	10.84	11.40
	Current **vs** RCP2.6-2070s	889.94	52.36	7.24	10.46
	Current **vs** RCP4.5-2070s	892.33	48.58	8.06	11.03
	Current **vs** RCP6.0-2070s	890.99	50.41	9.20	9.40
*Paeonia rockii* subsp.* taibaishanica*	Current **vs** RCP2.6-2050s	919.44	20.47	8.13	11.96
	Current **vs** RCP4.5-2050s	928.55	16.05	2.85	12.55
	Current **vs** RCP6.0-2050s	925.10	13.19	6.30	15.41
	Current **vs** RCP2.6-2070s	928.10	14.17	3.30	14.43
	Current **vs** RCP4.5-2070s	926.61	11.73	4.78	16.88
	Current **vs** RCP6.0-2070s	918.11	18.71	9.90	13.29

### Climatic niche comparisons

**Figure 7:**
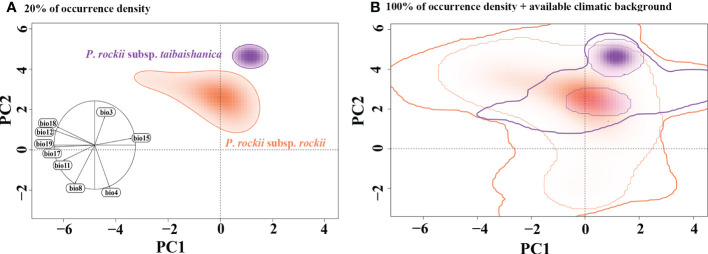
Global climatic space constructed over all background areas and realized niches of *P*. *rockii*, showing overlaps between *P*. *rockii* subsp. *rockii* and *P*.* rockii* subsp. *taibaishanica* distribution ranges, plotting a solid line representing the 20% of occurrence density **(A)**, and 100% of occurrence density with a thin line and 100% of available climatic background with a thick line. **(B)** The left graph includes the contribution and direction of each variable to the two-first components of the PCA-env.

Analysis based on the E-space niche model using PCA-env analysis showed that it accounted for 75.43% (PC1 = 52.22% and PC2 = 23.21%) of the total climatic variation in the areas occupied by the species and background areas (**Figure S9**). PC1 was associated with bio11 and bio8 as the main contributing variables, whereas bio12 and bio18 were mainly associated with PC2 (**Figure S9**). The multiple niche plot based on an occurrence density of displaying the 20% showed that the niche of *P*. *rockii* subsp. *rockii* was significantly differentiated from the niche of *P*. *rockii* subsp. *taibaishanica* (**Figure 7A**). When an occurrence density of 100% was plotted in the PCA-env space, high overlap was detected in the climatic space among ranges (**Figure 7B**). The climatic niche overlaps between the *P*. *rockii* subsp. *rockii* and *P*. *rockii* subsp. *taibaishanica* populations were characterized by (D = 0.062) the climatic niches changes to “unfilling” niche with 0.07, “stable” niche with 0.191, and “range expansion” niche with 0.809.

### Displacement trends based on the geometric centers of suitable habitats

**Figure 8:**
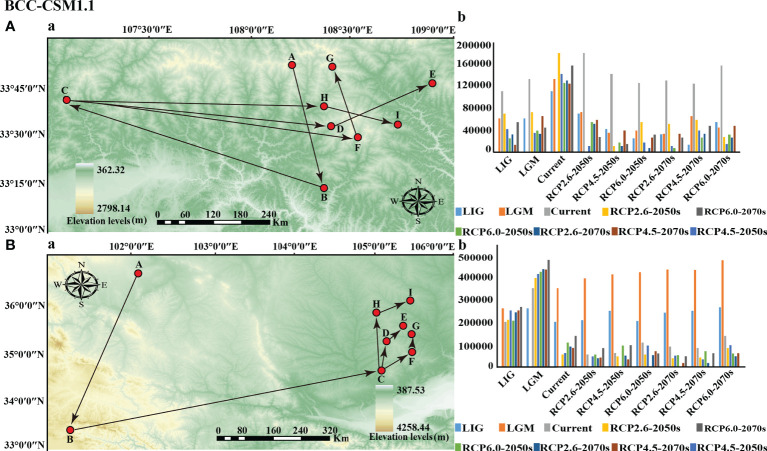
Migration of the center of suitable habitat for two subspecies of *P*. *rockii* since the last interglacial period (**A**: *P*. *rockii* subsp. *rockii*; **B**: *P*.* rockii* subsp. *taibaishanica*; (a) migration route; (b) migration distance). Among them, the meaning of the letters were (A) LIG, (B) LGM, (C) current, (D) RCP2.6-2050s, (E) RCP2.6-2070s, (F) RCP4.5-2050s, (G) RCP4.5-2070s, (H) RCP6.0-2050s, (I) RCP6.0-2070s.

The results of General Circulation Models (GCMs) showed that the directions and distances of the centroids of the suitable habitats for the two subspecies varied under different climate scenarios compared with the current period (**Figure 8**, **S10**). The current geometric center of the potentially suitable habitat for *P*. *rockii* subsp. *rockii* was located in Wuganyi Town, Liuba County, Shaanxi Province (107.063982 E, 33.556846 N). The BCC-CSM1.1 model indicates that by the 2050s, the centroids of the suitable areas for *P*. *rockii* subsp. *rockii* under the RCP 2.6, RCP 4.5, and RCP 6.0 climate scenarios are predicted to all move eastward to Chengguan Town, Taibaimiao Town, and Huanguan Town, Ningshan County, Shaanxi Province, with migration distances of 125.35 km, 138.73 km, and 121.49 km, respectively (**Figure 8A**). By the 2070s, the centroids of the suitable areas for *P*. *rockii* subsp. *rockii* is expected to migrate further to the northeast and reach Dongchuan Town, Zhen’an County, Huanguan Town, Ningshan County, and Yangsi Town, Zhen’an County, Shaanxi Province, with migration distances of 172.21 km, 125.98 km, and 156.32 km under RCP 2.6, RCP 4.5, and RCP 6.0, respectively (**Figure 8A**). At the same time, the BCC-CSM2-MR and MIROC5 models prediction indicate that the centroids of the suitable areas for *P*. *rockii* subsp. *rockii* is expected to migration direction be similar to the BCC-CSM1.1 results in two different time horizons (2050 and 2070) (**Figure S10**). The centroid of the suitable area for *P*. *rockii* subsp. *taibaishanica* at present was located at the junction of Longdong and Shaanxi. The BCC-CSM1.1 model demonstrated that by the 2050s, the centroids of the suitable areas for *P*. *rockii* subsp. *taibaishanica* under the RCP2.6, RCP 4.5, and RCP 6.0 climate scenarios are predicted to migrate to the northeast, with migration distances of 53.35 km, 59.59 km, and 103.05 km, respectively (**Figure 8B**). By the 2070s, the centroids of the suitable areas for *P*. *rockii* subsp. *taibaishanica* is expected to continue to migrate northward under the RCP2.6, RCP 4.5, and RCP 6.0 climate scenarios, with migration distances of 86.27 km, 80.76 km, and 131.04 km, respectively (**Figure 8B**). In addition, the BCC-CSM2-MR and MIROC5 model predictions suggest that the centroids of the suitable areas for *P*. *rockii* subsp. *taibaishanica* is expected to migrate to the northeast, at two different time horizons (2050 and 2070) (**Figure S10**).

## Discussion

### Impacts of climate change on spatial distributions of the two subspecies

At present, the potential highly suitable areas for *P*. *rockii* subsp. *rockii* and *P*. *rockii* subsp. *taibaishanica* are mainly concentrated in southern Gansu, Ziwu Ridge and the Qinling Mountains in Shaanxi, the Liupan Mountains in Ningxia, northern Chongqing, and northern Hubei (**Figures 5**, **S7**). The predicted results were consistent with the actual distributions, but the distribution areas were larger than the actual distribution areas. During the last glaciation, the global temperature was 5–12°C lower than now and the glaciated area was 8.4 times that at the present in China (Wang and Liu, 2001; Li et al., 2004), which profoundly affected the distributions of plants. However, our results showed that the suitable distribution areas for the two subspecies of *P*. *rockii* did not contract as expected during the LGM period (**Figures 5**, **S7**, and **Table 3**). By contrast, the suitable distribution areas decreased during the LIG, expanded during the LGM period. Range expansions during the LGM have also been found in other plant taxa, such as *Pseudotaxus chienii*, *Picea likiangensis* var. *likiangensis*, *Taxus wallichiana*, and *Tsuga dumosa* (Liu et al., 2013; Yu et al., 2015; Zhang et al., 2018; Zhang et al., 2020). Clearly the low temperatures in the ice age did not reduce in the ranges of all plants, but instead they provided suitable conditions for the expansion of some woody plants (Kozhoridze et al., 2015). The relatively low latitude of China and its complex topography and mountain barriers mean that China was not as heavily glaciated as Europe (Qiu et al., 2011). Therefore, some plants retreated southward and spread northward during the glacial period (Hewitt, 2000; Ravelo et al., 2004; Gratton et al., 2008).

Under future climate change scenarios, our results suggest that the suitable distribution areas for the two subspecies will tend to shrink in the future. Similar predictions have been made for other rare and endangered plants, such as *Metasequoia glyptostroboides*, *Cercidiphyllum japonicum*, and *Liriodendron chinense* (Lv, 2009; Zhu and Xu, 2019). Under the RCP6.0 scenario, the future temperature will increase by 1.3°C in 2050 and 2.2°C in 2070 (Ford et al., 2012). Global climate change will lead to melting of the snow caps, retreat of glaciers, increased rainfall, and intensification of drought conditions, thereby leading to changes in biological phenology, with fragmentation and loss of habitats for many species (Waldvogel et al., 2020). However, the suitable distribution of species can involve biological, geological or other disturbance factors in addition to the effects of climate change. Here, three plausible reasons may explain the suitable distribution areas of the two subspecies tends to shrink in the future. Firstly, it is mainly due to the human factor. The destructive excavation of residents in the distribution area frequently occurs, and the quantity of natural Moutan Cortex (Mudanpi in Chinese) for medicine has decreased and prices have soared, which leads to the destructive excavation of the older tree peony by the residents, and the regeneration of wild resources is difficult (Cui et al., 2021). Secondly, the species currently has a sporadic “island” distribution in the Qinling Mountains and Ziwu Ridge and their adjacent areas, as well as in southern Gansu (**Figure S1**). Recent studies have shown that wild ungulates, the development of tourism and residential infrastructure and recreational activities in protected areas have a negative impact on the distribution of species (Bárrios and Hamilton, 2021). So, we speculate that this may be one of the reasons why suitable habitat for the species is declining. Thirdly, the geological hazards common in mountainous areas (landslides and mudslides) may have a negative impact on the distribution of the species. As a result the characteristics of large mountainous areas and large relief in the Qinling Mountains and adjacent areas, a wide range of geological disasters have been formed, such as mudslides, collapses, and landslides. (Liu, 2005). They occurred not only in the high mountains and deep valleys of the western Qinling Mountains, but also in the low hills and basins of the eastern Qinling Mountains (Dong et al., 2022).

Under the influence of global climate change, similar changes occurred to the distribution ranges of the two subspecies. Simulation results from three high-resolution GCMs (BCC-CSM1.1, BCC-CSM2-MR and MIROC5) show that the centers of the suitable areas are predicted to tend to displacement toward high latitudes and elevations, under the emissions scenarios for the 2050s and 2070s (**Figure 8** and **S10**). The elevation of species distribution is largely driven by the temperature gradients, so as the climate warms, species ranges shift to higher altitudes (Lenoir et al., 2008; Osorio-Canadas et al., 2021). Indeed, numerous studies have demonstrated that climate change has altered the distribution patterns of species, where many species have moved to higher latitudes or higher altitudes (Parmesan and Yohe, 2003; Colwell et al., 2008). As our study demonstrates, ongoing climate change is altering the distribution of two subspecies and is more likely to face the risk of habitat losses, making conservation measures for two subspecies an urgent issue. For two subspecies, implementation of a conservation strategy based on population niche models may require *in situ* conservation, considering that most current suitable areas were predicted to still be suitable for two subspecies under climate change. Therefore, in order to avoid threats to the populations of the two subspecies from anthropogenic activities, set core protection areas at highly elevated regions of the Qinba Mountains and the Ziwu Ridge. In addition, in ex-situ conservation, mature seeds of each population can be collected, artificially planted in botanical gardens, and then transplanted into wild populations to strengthen gene exchange between populations and improve the level of genetic diversity of wild populations.

### Species divergence

Spatial interruptions provided by mountains have key effects on the morphology and divergence of species because topographic complexity leads to ecological stratification and environmental heterogeneity (Fjeldså et al., 2012). We found that Paeoniaceae separated from other members of the Saxifragales about 96.78 Ma in the Late Cretaceous according to BEAST analysis of whole plastid genomes (**Figure 3**), and this result is consistent with the date of 90–100 Ma obtained in previous dating analyses (Tank et al., 2015; Folk et al., 2019). The divergence of woody and herbaceous *Paeonia* species occurred in the Miocene Aquitanian at 20.78 Ma (95% HPD: 12.86–29.29 Ma). The divergence of sections *Moutan* and *Onaepia* occurred during the Miocene Serravallian at 13.44 Ma (95% HPD: 5.32–20.45 Ma). The other divergence events (with the exception of the split between *P*. *emodi* and other species in the section *Paeonia*) occurred during the following 9.34–0.66 Ma (**Figure 3**), i.e., within the Miocene Tortonian to Middle Pleistocene.

Environmental heterogeneity plays an important role in species differentiation and ecological adaptation (Huang et al., 2021). Niche differentiation will enhance the divergence of species following spatial isolation (Liu et al., 2013), and it may lead to different populations of the same species with different adaptations under different environmental conditions (Shen et al., 2010). We must be cautious when interpreting molecular dating results, but our estimated divergence times coincide with the period of strong uplift of the QTP (Sun et al., 2011). Thus, the current distribution of the two subspecies may be largely determined by the QTP uplift, which is thought to be related to past geographical and climatic fluctuations. Under the influence of the Himalayan orogeny, the QTP and Hengduan Mountains continued to rise, and various mountain uplift events resulted in high climate variability together with a gradual drop in temperature (Ye et al., 2016). The environmental climate shock may have played an important role in promoting the origin and divergence of *Paeonia* plants. In addition, niche models suggest that the two subspecies populations may occupy different climatic niches (**Figure 7A**), although some niches may overlap between the two subspecies based on simulation with 100% occurrence (**Figure 7B**). Furthermore, the E-space results suggest that annual precipitation (bio12), precipitation of warmest quarter (bio18) and mean temperature in the coldest season (bio11) were potential ecological factors associated with differences between the two subspecies (**Figure S9**). Climate seasonality (i.e. temperature and precipitation seasonality) is one of the key ecological factors affecting phenology (i.e. the timing of cyclical and seasonal natural phenomena such as flowering and defoliation) (Quintero et al., 2014). Thus, gene flow between populations of the two subspecies may be restricted due to the potential genetic barriers caused by the asynchronous phenology, leading to population differentiation.

### Phylogenetic relationships

RAxML analysis showed that the topologies of the three different data sets were identical and all of the nodes had high bootstrap values (**Figures 2**, **S3**, **S4**). The results showed that the Paeoniaceae species clustered into a clade, which was further divided into sections *Paeonia*, *Moutan* and *Onaepia*, where the sections *Moutan*, and *Onaepia* were grouped into a large evolutionary lineage with high bootstrap support (**Figures 2**, **S3**, **S4**). These findings are consistent with previous studies based on morphological taxonomy and molecular phylogenetics in *Paeonia* (Hong et al., 2001; Zhang et al., 2020; Wu et al., 2021; Zhou et al., 2021). We also analyzed the interspecific affinities and found that the species in section *Moutan* section were divided into two subsections. *P*. *ludlowii* and *P*. *delavayi* var. *lutea* clustered together at the base of the section *Moutan* branch. *P*. *jishanensis*, *P*. *decomposita*, *P*. *qiui*, *P*. *suffruticosa*, *P*. *ostii*, *P*. *rockii* subsp. *taibaishanica*, and *P*. *rockii* subsp. *rockii* clustered together. *P*. *rockii* subsp. *rockii* and *P*. *rockii* subsp. *taibaishanica* have very similar morphological characters (e.g., white petals with a large, dark purple spot at base; disk wholly enveloping carpels, pale yellow, leathery, apex dentate, or lobed) and they formed a clade (bootstrap support = 100%) in the phylogenetic trees constructed from the WP data set, PCG data set, and the GBDN data set, which is consistent with taxonomic evidence from previous studies (Hong et al., 2001).

## Conclusions

In this study, we used whole chloroplast genomes and niche analyses to investigate the phylogenetic relationships, species divergence and demographic history of *Paeonia rockii*, which is rare and endangered medicinal plants in East Asia. The phylogenetic results showed that *P*. *rockii* subsp. *rockii* was most closely to *P*. *rockii* subsp. *taibaishanica* and was grouped into a single branch, with an estimated divergence time of approximately 0.83 million years ago (Ma). In addition, ecological niche analyses indicated that the potential habitat of two subspecies may displacement northeastward in response to 21st century global climate change. Indeed, *P*. *rockii*, as a rare and endangered species in East Asia, is a vital representative of its community structure. The results in this study may provide insights into the protectection and utilization of the endangered *P*. *rockii* species. Further, they may play an important role in exploring the population dynamics of other rare and endangered tree species, while providing a scientific basis for understanding the evolutionary history and ecological adaptation of rare and endangered species in East Asia.

## Data availability statement

The data presented in the study are deposited in the National Center for Biotechnology Information repository, accession number OK235337, MW192444.

## Author contributions

YC conceived and coordinated the study. P-BD and L-JW analyzed the data and performed bioinformatics analyses. YC, F-XG, P-BD, L-JW, YJ, Z-HL and H-YW provided some materials and analytical tools. YC, F-XG, P-BD, YJ, and L-JW wrote and revised the manuscript. All authors contributed to the article and approved the submitted version.

## Funding

This study was financially supported by the Research on breeding, cultivation, storage and processing of traditional Chinese medicinal materials in Beishan, Yuzhong (70103619011), a study on the mechanism of growth age on seed quality and production performance of *Astragalus membranaceus* (22JR5RA846).

## Conflict of interest

The authors declare that the research was conducted in the absence of any commercial or financial relationships that could be construed as a potential conflict of interest.

## Publisher’s note

All claims expressed in this article are solely those of the authors and do not necessarily represent those of their affiliated organizations, or those of the publisher, the editors and the reviewers. Any product that may be evaluated in this article, or claim that may be made by its manufacturer, is not guaranteed or endorsed by the publisher.

## Supplementary material

Supplementary Figure 1 Geographical distribution of *Paeonia rockii*.

Supplementary Figure 2 Chloroplast genome maps of two subspecies.

Supplementary Figure 3 The maximum likelihood (ML) tree of Paeoniaceae inferred from the protein-coding genes data set. Numbers at nodes correspond to ML bootstrap percentages (100 replicates, only values greater than 50% are shown).

Supplementary Figure 4 The maximum likelihood (ML) tree of Paeoniaceae inferred from remove the ambiguously aligned sites in the whole plastid genome data set. Numbers at nodes correspond to ML bootstrap percentages (100 replicates, only values greater than 50% are shown).

Supplementary Figure 5 Boxplots of nine climatic variables selected for two subspecies of *P*. *rockii*.

Supplementary Figure 6 Environmental variables contributions of the MAXENT model at present for two subspecies of *P*. *rockii*.

Supplementary Figure 7 Potential distribution for *P*.* rockii* subsp.* taibaishanica* during different periods predicted by the MaxEnt model based on optimized parameters. The potential distribution of *P*. *rockii* subsp. *rockii* was divided into four grades by the natural breaks method. Gray, green, yellow, and red areas represent not suitable, marginally suitable, moderately suitable, and highly suitable areas, respectively.

Supplementary Figure 8 Potential distribution for *P*.* rockii* subsp. *taibaishanica* during different periods predicted by the MaxEnt model based on optimized parameters. The potential distribution of *P*. *rockii* subsp. *rockii* was divided into four grades by the natural breaks method. Gray, green, yellow, and red areas represent not suitable, marginally

Supplementary Figure 9 Global climatic space represented through a PCA-env (explaining PC1 = 52.22% and PC2 = PC2 = 23.21% of the total climatic variation).

Supplementary Figure 10 Migration of the center of suitable habitat for two subspecies of *P*. *rockii* since the last interglacial period (a. migration route; b. migration distance). Among them, the meaning of the letters were **(A)** LIG, **(B)** LGM, **(C)** current, **(D)** RCP2.6-2050s, **(E)** RCP2.6-2070s, **(F)** RCP4.5-2050s, **(G)** RCP4.5-2070s, **(H)** RCP6.0-2050s, **(I)** RCP6.0-2070s.

## References

[B1] Akiyama R. Sun J. Q. Hatakeyama M. L. Lischer H. E. Briskine R. V. Hay A.. (2020). Fine-scale empirical data on niche divergence and homeolog expression patterns in an allopolyploid and its diploid progenitor species. New Phytol. 229, 3587–3601. doi: 10.1111/nph.17101 33222195 PMC7986779

[B2] Baniaga A. E. Marx H. E. Arrigo N. Barker M. S. (2020). Polyploid plants have faster rates of multivariate niche differentiation than their diploid relatives. Ecol. Lett. 23, 68–78. doi: 10.1111/ele.13402 31637845

[B3] Bankevich A. Nurk S. Antipov D. Gurevich A. A. Dvorkin M. Kulikov A. S.. (2012). SPAdes: a new genome assembly algorithm and its applications to single-cell sequencing. J. Comput. Biol. 19, 455–477. doi: 10.1089/cmb.2012.0021 22506599 PMC3342519

[B4] Bárrios S. Hamilton M. A. (2021). Conservation status of native plant hybrids in the British virgin islands. Biodivers. Data J. 9, e62809. doi: 10.3897/BDJ.9.e62809 33776530 PMC7987705

[B5] Bates O. K. Ollier S. Bertelsmeier C. (2020). Smaller climatic niche shifts in invasive than non-invasive alien ant species. Nat. Commun. 11, 1–8. doi: 10.1038/s41467-020-19031-1 33060612 PMC7567077

[B6] Broennimann O. Fitzpatrick M. C. Pearman P. B. Petitpierre B. Pellissier L. Yoccoz N. G.. (2011). Measuring ecological niche overlap from occurrence and spatial environmental. Global Ecol. Biogeogr. 21, 481–497. doi: 10.1111/j.1466-8238.2011.00698.x

[B7] Chen I. C. Hill J. K. Ohlemüller R. Roy D. B. Thomas C. D. (2011). Rapid range shifts of species associated with high levels of climate warming. Science 333, 1024–1026. doi: 10.1126/science.1206432 21852500

[B8] Chen Q. Yin Y. Zhao R. Yang Y. Teixeira da Silva J. A. Yu X. (2020). Incorporating local adaptation into species distribution modeling of *Paeonia mairei*, an endemic plant to China. Front. Plant Sci. 10. doi: 10.3389/fpls.2019.01717 PMC6997482 32047503

[B9] Chumley T. W. Palmer J. D. Mower J. P. Fourcade H. M. Calie P. J. Boore J. L.. (2006). The complete chloroplast genome sequence of pelargonium × hortorum: organization and evolution of the largest and most highly rearranged chloroplast genome of land plants. Mol. Biol. Evol. 23, 2175–2190. doi: 10.1093/molbev/msl089 16916942

[B10] Clowers K. J. Will J. L. Gasch A. P. (2015). A unique ecological niche fosters hybridization of oak-tree and vineyard isolates of saccharomyces cerevisiae. Mol. Ecol. 24, 5886–5898. doi: 10.1111/mec.13439 26518477 PMC4824287

[B11] Cola V. D. Broennimann O. Petitpierre B. Breiner F. T. D'Amen M. Randin C.. (2017). Ecospat: an r package to support spatial analyses and modeling of species niches and distributions. Ecography 40, 774–787. doi: 10.1111/ecog.02671

[B12] Colwell R. K. Brehm G. Cardelús C. L. Gilman A. C. Longino J. T. (2008). Global warming, elevational range shifts, and lowland biotic attrition in the wet tropics. Science 322, 258–261. doi: 10.1126/science.1162547 18845754

[B13] Cui Z. J. Ma Y. Z. Jin L. Ma Y. Wang Z. H. Liu L. (2021). New wild populations of three rare and endangered species discovered in fourth survey of traditional chinese medicine resources in gansu province. Mod. Chin. Med. 23, 1168–1171. doi: 10.13313/j.issn.1673-4890.20200914008

[B14] Darriba D. Taboada G. L. Doallo R. Posada D. (2012). jModelTest 2: more models, new heuristics and parallel computing. Nat. Methods 9, 772–772. doi: 10.1038/nmeth.2109 PMC4594756 22847109

[B15] Daskalova G. N. Myers-Smith I. H. Godlee J. L. (2020). Rare and common vertebrates span a wide spectrum of population trends. Nat. Commun. 11, 1–13. doi: 10.1038/s41467-020-17779-0 32879314 PMC7468135

[B16] Dong Y. Shi X. Sun S. Sun J. Hui B. He D.. (2022). Co-Evolution of the Cenozoic tectonics, geomorphology, environment and ecosystem in the qinling mountains and adjacent areas, central China. Geosystems Geoenvironment. 1, 100032. doi: 10.1016/j.geogeo.2022.100032

[B17] Doyle J. J. Doyle J. L. (1987). A rapid DNA isolation procedure for small quantities of fresh leaf tissue. Phytochem. Bull. 19, 11–15.

[B18] Drouin G. Daoud H. Xia J. (2008). Relative rates of synonymous substitutions in the mitochondrial, chloroplast and nuclear genomes of seed plants. Mol. Phylogenet. Evol. 49, 827–831. doi: 10.1016/j.ympev.2008.09.009 18838124

[B19] Drummond A. J. Suchard M. A. Xie D. Rambaut A. (2012). Bayesian Phylogenetics with BEAUti and the BEAST 1.7. Mol. Biol. Evol. 29, 1969–1973. doi: 10.1093/molbev/mss075 22367748 PMC3408070

[B20] Fjeldså J. Bowie R. C. K. Rahbek C. (2012). The role of mountain ranges in the diversification of birds. Annu. Rev. Ecol. Evol. S. 43, 249–265. doi: 10.1146/annurev-ecolsys-102710-145113

[B21] Folk R. A. Stubbs R. L. Mort M. E. Cellinese N. Allen J. M. Soltis P. S.. (2019). Rates of niche and phenotype evolution lag behind diversification in a temperate radiation. P. Natl. A. Sci. India. B. 116, 10874–10882. doi: 10.1073/pnas.1817999116 PMC6561174 31085636

[B22] Ford J. D. Vanderbilt W. Berrang-Ford L. (2012). Authorship in IPCC AR5 and its implications for content: climate change and indigenous populations in WGII. Climatic Change 113, 201–213. doi: 10.1007/s10584-011-0350-z 26005230 PMC4439732

[B23] Gang G. H. Cho G. Kwak Y. S. Park E. H. (2017). Distribution of rhizosphere and endosphere fungi on the first-class endangered plant *Cypripedium japonicum* . Mycobiology 45, 97–100. doi: 10.5941/MYCO.2017.45.2.97 28781542 PMC5541154

[B24] Garza G. Rivera A. Venegas Barrera C. S. Martinez-Ávalos J. G. Dale J. Feria Arroyo T. P. (2020). Potential effects of climate change on the geographic distribution of the endangered plant species manihot walkerae. Forests 11, 689–704. doi: 10.3390/f11060689

[B25] Gratton P. Konopiński M. K. Sbordoni V. (2008). Pleistocene evolutionary history of the clouded Apollo (*Parnassius mnemosyne*): genetic signatures of climate cycles and a ‘time-dependent’ mitochondrial substitution rate. Mol. Ecol. 17, 4248–4262. doi: 10.1111/j.1365-294X.2008.03901.x 18986502

[B26] Hebbar K. B. Abhin P. S. Sanjo Jose V. Neethu P. Santhosh A. Shil S.. (2022). Predicting the potential suitable climate for coconut (*Cocos nucifera* l.) cultivation in India under climate change scenarios using the MaxEnt model. Plants 11, 731–754. doi: 10.3390/plants11060731 35336613 PMC8954727

[B27] He D. Y. Dai S. M. (2011). Anti-inflammatory and immunomodulatory effects of paeonia lactiflora pall., a traditional Chinese herbal medicine. Front. Pharmacol. 2. doi: 10.3389/fphar.2011.00010 PMC3108611 21687505

[B28] Hermsen E. J. Gandolfo M. A. Nixon K. C. Crepet W. L. (2003). Divisestylus gen. nov.(aff. iteaceae), a fossil saxifrage from the late Cretaceous of new Jersey, USA. Am. J. Bot. 90, 1373–1388. doi: 10.3732/ajb.90.9.1373 21659237

[B29] Herrando-Moraira S. Nualart N. Herrando-Moraira A. Chung M. Y. Chung M. G. López-Pujol J. (2019). Climatic niche characteristics of native and invasive *Lilium lancifolium* . Sci. Rep-UK 9, 1–16. doi: 10.1038/s41598-019-50762-4 PMC6778149 31586099

[B30] Hewitt G. (2000). The genetic legacy of the quaternary ice ages. Nature 405, 907–913. doi: 10.1038/35016000 10879524

[B31] Hijmans R. J. Cameron S. E. Parra J. L. Jones P. G. Jarvis A. (2005). Very high resolution interpolated climate surfaces for global land areas. Int. J. Climatol. 25, 1965–1978. doi: 10.1002/joc.1276

[B32] Hong D. Y. (2010). Peonies of the world: Taxonomy and phytogeography[M] (Kew Publishing: Royal Botanic Gardens).

[B33] Hong D. Y. Pan K. Y. J.Turland. N. (2001). “Paeoniaceae,” in Flora of China, vol. 6 . Eds. Wu Z. Y. Raven P. H. (Beijing: Science Press), 127–132.

[B34] Huang R. Xie X. Chen A. Li F. Tian E. Chao Z. (2021). The chloroplast genomes of four bupleurum (Apiaceae) species endemic to southwestern China, a diversity center of the genus, as well as their evolutionary implications and phylogenetic inferences. BMC Genomics 22, 1–15. doi: 10.1186/s12864-021-08008-z 34600494 PMC8487540

[B35] Jeffroy O. Brinkmann H. Delsuc F. Philippe H. (2006). Phylogenomics: the beginning of incongruence? Trends Genet. 22, 225–231. doi: 10.1016/j.tig.2006.02.003 16490279

[B36] Jin J. J. Yu W. B. Yang J. B. Song Y. Yi T. S. Li D. Z. (2018). GetOrganelle: a simple and fast pipeline for *de novo* assembly of a complete circular chloroplast genome using genome skimming data. *Bio* . Rxiv. 4, 256479–256488. doi: 10.1101/256479

[B37] Johnson E. E. Escobar L. E. Zambrana-Torrelio C. (2019). An ecological framework for modeling the geography of disease transmission. Trends. Ecol. Evol. 34, 655–668. doi: 10.1016/j.tree.2019.03.004 31078330 PMC7114676

[B38] Jump A. S. Penuelas J. (2005). Running to stand still: adaptation and the response of plants to rapid climate change. Ecol. Lett. 8, 1010–1020. doi: 10.1111/j.1461-0248.2005.00796.x 34517682

[B39] Katoh K. Kuma K. I. Toh H. Miyata T. (2005). MAFFT version 5: improvement in accuracy of multiple sequence alignment. Nucleic Acids Res. 33, 511–518. doi: 10.1093/nar/gki198 15661851 PMC548345

[B40] Kearse M. Moir R. Wilson A. Stones-Havas S. Cheung M. Sturrock S.. (2012). Geneious basic: an integrated and extendable desktop software platform for the organization and analysis of sequence data. Bioinformatics 28, 1647–1649. doi: 10.1093/bioinformatics/bts199 22543367 PMC3371832

[B41] Koebsch F. Sonnentag O. Järveoja J. Peltoniemi M. Alekseychik P. Aurela M.. (2020). Refining the role of phenology in regulating gross ecosystem productivity across European peatlands. Global Change Biol. 26, 876–887. doi: 10.1111/gcb.14905 31686431

[B42] Kozhoridze G. Orlovsky N. Orlovsky L. Blumberg D. G. Golan-Goldhirsh A. (2015). Geographic distribution and migration pathways of pistacia-present, past and future. Ecography 38, 1–14. doi: 10.1111/ecog.01496

[B43] Krzywinski M. Schein J. Birol I. Connors J. Gascoyne R. Horsman D.. (2009). Circos: an information aesthetic for comparative genomics. Genome Res. 19, 1639–1645. doi: 10.1101/gr.092759.109 19541911 PMC2752132

[B44] Kumar S. Stecher G. Li M. Knyaz C. Tamura K. (2018). MEGA X: molecular evolutionary genetics analysis across computing platforms. Mol. Biol. Evol. 35, 1547–1549 doi: 10.1093/molbev/msy096 29722887 PMC5967553

[B45] Lee S. R. Choi J. E. Lee B. Y. Yu J. N. Lim C. E. (2018). Genetic diversity and structure of an endangered medicinal herb: implications for conservation. AoB Plants 10, ply021–ply030. doi: 10.1093/aobpla/ply021 29692882 PMC5909456

[B46] Lehikoinen P. Santangeli A. Jaatinen K. Rajasärkkä A. Lehikoinen A. (2019). Protected areas act as a buffer against detrimental effects of climate change-evidence from large-scale, long-term abundance data. Global Change Biol. 25, 304–313. doi: 10.1111/gcb.14461 30393928

[B47] Lehikoinen A. Virkkala R. (2016). North by north-west: Climate change and directions of density shifts in birds. Global Change Biol. 22, 1121–1129. doi: 10.1111/gcb.13150 26691578

[B48] Lenoir J. Gegout J. C. Marquet P. A. de Ruffray P. Brisse H. (2008). A signifificant upward shift in plant species optimum elevation during the 20th century. Science 320, 1768–1771. 1768 doi: 10.1126/science.1156831 18583610

[B49] Li K. R. (1996). Research progress of global climate change and its impact and future prospect. Acta Geographica Sinica. S1, 1–14.

[B50] Li L. Chen J. K. (2014). Influence of climate change on wild plants and the conservation strategies. Biodiversity Science. 22, 549–563. doi: 10.3724/SP.J.1003.2014.14124

[B51] Li J. J. Shu Q. Zhou S. Z. Zhao Z. J. Zhang J. M. (2004). Review and prospects of quaternary glaciation research in China. J. Glaciol Geocryol. 26, 235–243.

[B52] Liu H. J. (2005). Uplift and the environmental disastrous effects in qinling mountains. Northwestern Geology 38, 89–93.

[B53] Liu J. Möller M. Provan J. Gao L. M. Poudel R. C. Li D. Z. (2013). Geological and ecological factors drive cryptic speciation of yews in a biodiversity hotspot. New Phytol. 199, 1093–1108. doi: 10.1111/nph.12336 23718262

[B54] Lv J. J. (2009). The impacts of climate change on the distribution of rare of endangered species in China and adaptation strategies D (Chinese Research Academy of Environmental Sciences).

[B55] Mader M. Schroeder H. Schott T. Schöning-Stierand K. Leite Montalvão A. P. Liesebach H.. (2020). Mitochondrial genome of *Fagus sylvatica* l. as a source for taxonomic marker development in the fagales. Plants. 9, 1274–1293. doi: 10.3390/plants9101274 PMC7650814 32992588

[B56] Moreno R. Zamora R. Molina J. R. Vasquez A. Herrera M.Á. (2011). Predictive modeling of microhabitats for endemic birds in south Chilean temperate forests using maximum entropy (Maxent). Ecol. Inform. 6, 364–370. doi: 10.1016/j.ecoinf.2011.07.003

[B57] Osorio-Canadas S. Flores-Hernández N. Sánchez-Ortiz T. Valiente-Banuet A. (2021). Changes in the structure and composition of the ‘Mexical’scrubland bee community along an elevational gradient. PLoS One 16, 1–22. doi: 10.1371/journal.pone.0254072 PMC8248643 34197555

[B58] Parmesan C. Yohe G. (2003). A globally coherent fingerprint of climate change impacts across natural systems. Nature 421, 37–42. doi: 10.1038/nature01286 12511946

[B59] Patel R. K. Jain M. (2012). NGS QC toolkit: a toolkit for quality control of next generation sequencing data. PLoS One 7, e30619–e30625. doi: 10.1371/journal.pone.0030619 22312429 PMC3270013

[B60] Peng S. J. Liu Y. P. Lyu T. Zhang X. L. Li Y. Q. Wang Z. H. (2021). Towards an understanding of the latitudinal patterns in thermal tolerance and vulnerability of woody plants under climate warming. Ecography 44, 1797–1807. doi: 10.1111/ecog.05582

[B61] Phillips S. J. Anderson R. P. Dudík M. Schapire R. E. Blair M. E. (2017). Opening the black box: An open-source release of maxent. Ecography 40, 887–893. doi: 10.1111/ecog.03049

[B62] Qiu Y. X. Fu C. X. Comes H. P. (2011). Plant molecular phylogeography in China and adjacent regions: tracing the genetic imprints of quaternary climate and environmental change in the world’s most diverse temperate flora. Mol. Phylogenet Evol. 59, 225–244. doi: 10.1016/j.ympev.2011.01.012 21292014

[B63] Quintero I. Gonzalez-Caro S. Zalamea P. C. Cadena C. D. (2014). Asynchrony of seasons genetic difffferentiation associated with geographic variation in climatic seasonality and reproductive phenology. Am. Nat. 184, 352–363. doi: 10.1086/677261 25141144

[B64] Qu X. J. Moore M. J. Li D. Z. Yi T. S. (2019). PGA: a software package for rapid, accurate, and flexible batch annotation of plastomes. Plant Methods 15, 1–12. doi: 10.1186/s13007-019-0435-7 31139240 PMC6528300

[B65] Ravelo A. C. Andreasen D. H. Lyle M. Lyle A. O. Wara M. W. (2004). Regional climate shifts caused by gradual global cooling in the pliocene epoch. Nature 429, 263–267. doi: 10.1038/nature02567 15152244

[B66] Rebelo H. Tarroso P. Jones G. (2010). Predicted impact of climate change on European bats in relation to their biogeographic patterns. Global Change Biol. 16, 561–576. doi: 10.1111/j.1365-2486.2009.02021.x

[B67] Renwick K. M. Curtis C. Kleinhesselink A. R. Schlaepfer D. Bradley B. A. Aldridge C. L.. (2018). Multi-model comparison highlights consistency in predicted effect of warming on a semi-arid shrub. Global Change Biol. 24, 424–438. doi: 10.1111/gcb.13900 28895271

[B68] Rhoné B. Defrance D. Berthouly-Salazar C. Mariac C. Cubry P. Couderc M.. (2020). Pearl millet genomic vulnerability to climate change in West Africa highlights the need for regional collaboration. Nat. Commun. 11, 1–9. doi: 10.1038/s41467-020-19066-4 33077747 PMC7573578

[B69] Rutledge D. (2011). Estimating long-term world coal production with logit and probit transforms. Int. J. Coal. Geol. 85, 23–33. doi: 10.1016/j.coal.2010.10.012

[B70] Shen J. W. Pike D. A. Du W. G. (2010). Movements and microhabitat use of translocated big-headed turtles (Platysternon megacephalum) in southern China. Chelonian Conserv. Bi. 9, 154–161. doi: 10.2744/CCB-0833.1

[B71] Solomon S. D. Qin D. Manning M. Averyt K. Marquis M. (Eds.). (2007). Climate change 2007-the physical science basis: Working Group I Contribution to Fourth Assess. Rep. IPCC. Vol. 4. Cambridge University Press.

[B72] Stamatakis A. (2006). RAxML-VI-HPC: maximum likelihood-based phylogenetic analyses with thousands of taxa and mixed models. Bioinformatics 22, 2688–2690. doi: 10.1093/bioinformatics/btl446 16928733

[B73] Sun B. N. Wu J. Y. Liu Y. S. Ding S. T. Li X. C. Xie S. P.. (2011). Reconstructing neogene vegetation and climates to infer tectonic uplift in western yunnan, China. Palaeogeogr. Palaeocl. 304, 328–336. doi: 10.1016/j.palaeo.2010.09.023

[B74] Talavera G. Castresana J. (2007). Improvement of phylogenies after removing divergent and ambiguously aligned blocks from protein sequence alignments. Syst. Biol. 56, 564–577. doi: 10.2307/20143065 17654362

[B75] Tank D. C. Eastman J. M. Pennell M. W. Soltis P. S. Soltis D. E. Hinchliff C. E.. (2015). Nested radiations and the pulse of angiosperm diversification: increased diversification rates often follow whole genome duplications. New Phytol. 207, 454–467. doi: 10.1111/nph.13491 26053261

[B76] Thomas C. D. Cameron A. Green R. E. Bakkenes M. Beaumont L. J. Collingham Y. C.. (2004). Extinction risk from climate change. Nature 427, 145–148. doi: 10.1038/nature02121 14712274

[B77] Waldvogel A. M. Feldmeyer B. Rolshausen G. Exposito-Alonso M. Rellstab C. Kofler R.. (2020). Evolutionary genomics can improve prediction of species’responses to climate change. Evol. Lett. 4, 4–18. doi: 10.1002/evl3.154 32055407 PMC7006467

[B78] Walther G. R. Gritti E. S. Berger S. Hickler T. Tang Z. Sykes M. T. (2007). Palms tracking climate change. Global Ecol. Biogeogr. 16, 801–809. doi: 10.1111/j.1466-8238.2007.00328.x

[B79] Wang S. Q. (2020). Genetic diversity and population structure of the endangered species paeonia decomposita endemic to China and implications for its conservation. BMC Plant Biol. 20, 1–14. doi: 10.1186/s12870-020-02682-z 33167894 PMC7650209

[B80] Wang J. L. Feng L. Y. Tang X. Bentley Y. Höök M. (2017). The implications of fossil fuel supply constraints on climate change projections: A supply-side analysis. Futures 86, 58–72. doi: 10.1016/j.futures.2016.04.007

[B81] Wang Z. T. Liu C. H. (2001). Geographical characteristics of the distribution of glaciers in China. J. Glaciol Geocryol. 23, 231–237.

[B82] Warren D. L. Glor R. E. Turelli M. (2008). Environmental niche equivalency versus conservatism: quantitative approaches to niche evolution. Evolution 62, 2868–2883. doi: 10.1111/j.1558-5646.2008.00482.x 18752605

[B83] Wick R. R. Schultz M. B. Zobel J. Holt K. E. (2015). Bandage: interactive visualization of *de novo* genome assemblies. Bioinformatics 31, 3350–3352. doi: 10.1093/bioinformatics/btv383 26099265 PMC4595904

[B84] Wu L. W. Nie L. P. Wang Q. Xu Z. C. Wang Y. He C. N.. (2021). Comparative and phylogenetic analyses of the chloroplast genomes of species of paeoniaceae. Sci. Rep-UK. 11, 1–16. doi: 10.1038/s41598-021-94137-0 PMC8289817 34282194

[B85] Wyman S. K. Jansen R. K. Boore J. L. (2004). Automatic annotation of organellar genomes with DOGMA. Bioinformatics 20, 3252–3255. doi: 10.1093/bioinformatics/bth352 15180927

[B86] Ye Z. Chen P. P. Bu W. J. (2016). Terrestrial mountain islands and pleistocene climate fluctuations as motors for speciation: a case study on the genus pseudovelia (Hemiptera: Veliidae). Sci. Rep-UK. 6, 1–12. doi: 10.1038/srep33625 PMC5030487 27650911

[B87] Yu H. B. Zhang Y. L. Liu L. S. Qi W. Li S. C. Hu Z. J. (2015). Combining the least cost path method with population genetic data and species distribution models to identify landscape connectivity during the late quaternary in Himalayan hemlock. Ecol. Evol. 5, 5781–5791. doi: 10.1002/ece3.1840 26811753 PMC4717335

[B88] Zhang M. H. Feng L. Zhu M. M. Gu J. F. Jiang J. Cheng X. D.. (2014). The anti-inflammation effect of moutan cortex on advanced glycation end products-induced rat mesangial cells dysfunction and highglucose-fat diet and streptozotocin-induced diabetic nephropathy rats. J. Ethnopharmacol. 151, 591–600. doi: 10.1016/j.jep.2013.11.015 24269777

[B89] Zhang W. X. Kou Y. X. Zhang L. Zeng W. D. Zhang Z. Y. (2020). Suitable distribution of endangered species *Pseudotaxus chienii* (Cheng) Cheng (Taxaceae) in five periods using niche modeling. Chin. J. Ecology. 39, 600–613. doi: 10.13292/j.1000-4890.202002.028

[B90] Zhang A. P. Wang Y. Xiong Q. L. Wu X. G. Sun X. M. Huang Y. M.. (2018). Distribution changes and refugia of three spruce taxa since the last interglacial. Chin. J. Appl. Ecol. 29, 2411–2421. doi: 10.13287/j.1001-9332.201807.027 30039681

[B91] Zhou Z. K. Crepet W. L. Nixon K. C. (2001). The earliest fossil evidence of the hamamelidaceae: Late Cretaceous (Turonian) inflorescences and fruits of altingioideae. Am. J. Bot. 88, 753–766. doi: 10.2307/2657028 11353701

[B92] Zhou S. L. Xu C. Liu J. Yu Y. Wu P. Cheng T.. (2021). Out of the pan-himalaya: Evolutionary history of the paeoniaceae revealed by phylogenomics. J. Syst. Evol. 59, 1170–1182. doi: 10.1111/jse.12688

[B93] Zhu Y. Y. Xu X. T. (2019). Effects of climate change on the distribution of wild population of metasequoia glyptostroboides, an endangered and endemic species in China. Chin. J. Ecology. 38, 1629–1636. doi: 10.13292/j.1000-4890.201906.018

