# Supplementary material for: Niche divergence at the intraspecific level in an endemic rare peony (Paeonia rockii): A phylogenetic, climatic and environmental survey
Source: Front Plant Sci. 2022 Nov 1;13:978011. doi: 10.3389/fpls.2022.978011 (PMC9663928; doi:10.3389/fpls.2022.978011)
Supplement: Supplementary Figure 1 — Geographical distribution of Paeonia rockii. [file DataSheet_1.zip › supplementary materials/Table S1.docx]

**Table S1** The samples information

| **No.** | **Species** | **GenBank number (cp)** | **Longitude** | **Latitude** |
| --- | --- | --- | --- | --- |
| 1 | *Paeonia rockii* subsp. *rockii* |  | 108.6463469 | 33.91364694 |
| 2 | *Paeonia rockii* subsp. *taibaishanica* |  | 107.694897 | 34.0645265 |
