# Supplementary material for: Niche divergence at the intraspecific level in an endemic rare peony (Paeonia rockii): A phylogenetic, climatic and environmental survey
Source: Front Plant Sci. 2022 Nov 1;13:978011. doi: 10.3389/fpls.2022.978011 (PMC9663928; doi:10.3389/fpls.2022.978011)
Supplement: Supplementary Figure 1 — Geographical distribution of Paeonia rockii. [file DataSheet_1.zip › supplementary materials/Table S2.docx]

**Table S2** Species NCBI sequence number information

| **No** | **Species** | **GenBank number (cp)** |
| --- | --- | --- |
| 1 | *Corylopsis spicata* | MK942341 |
| 2 | *Disanthus cercidifolius* subsp. *longipes* | NC_050371 |
| 3 | *Hamamelis mollis* | NC_037881 |
| 4 | *Rhodoleia championii* | NC_045276 |
| 5 | *Sinowilsonia henryi* | NC_036069 |
| 6 | *Paeonia anomala* | MT210549 |
| 7 | *Paeonia brownii* | JQ952560 |
| 8 | *Paeonia decomposita* | NC_039425 |
| 9 | *Paeonia delavayi* var. *lutea* | MT210546 |
| 10 | *Paeonia emodi* | MT210548 |
| 11 | *Paeonia intermedia* | MT210547 |
| 12 | *Paeonia jishanensis* | MT210545 |
| 13 | *Paeonia lactiflora* | MK860971 |
| 14 | *Paeonia ludlowii* | NC_035623 |
| 15 | *Paeonia obovata* | JQ952561 |
| 16 | *Paeonia ostii* | MK701990 |
| 17 | *Paeonia qiui* | MT210544 |
| 18 | *Paeonia suffruticosa* | JQ952559 |
| 19 | *Paeonia veitchii* | NC_032401 |
