# Supplementary material for: Niche divergence at the intraspecific level in an endemic rare peony (Paeonia rockii): A phylogenetic, climatic and environmental survey
Source: Front Plant Sci. 2022 Nov 1;13:978011. doi: 10.3389/fpls.2022.978011 (PMC9663928; doi:10.3389/fpls.2022.978011)
Supplement: Supplementary Figure 1 — Geographical distribution of Paeonia rockii. [file DataSheet_1.zip › supplementary materials/Table S3.docx]

**Table S3** Features of plastid genomes in 16 *Paeonia* samples.

| **Species** | **Size (bp)** | **LSC (bp)** | **SSC (bp)** | **IR (bp)** | **Number of total genes** | **Number of PCGs** | **Number of tRNA genes** | **Number of rRNA genes** | **GenBank number (cp)** |
| --- | --- | --- | --- | --- | --- | --- | --- | --- | --- |
| *Paeonia anomala* | 152,745 | 84,402 | 17,025 | 25,659 | 131 | 84 | 37 | 8 | MT210549 |
| *Paeonia brownii* | 152,226 | 84,261 | 16,679 | 25,643 | 120 | 83 | 36 | 8 | JQ952560 |
| *Paeonia decomposita* | 152,601 | 84,263 | 17,036 | 25,650 | 133 | 84 | 40 | 8 | NC_039425 |
| *Paeonia delavayi* var. *lutea* | 152,790 | 84,462 | 17,032 | 25,648 | 131 | 83 | 37 | 8 | MT210546 |
| *Paeonia emodi* | 152,828 | 84,465 | 17,001 | 25,681 | 131 | 82 | 37 | 8 | MT210548 |
| *Paeonia intermedia* | 152,713 | 84,333 | 17,024 | 25,678 | 131 | 84 | 37 | 8 | MT210547 |
| *Paeonia jishanensis* | 152,631 | 84,295 | 17,046 | 25,645 | 131 | 84 | 37 | 8 | MT210545 |
| *Paeonia lactiflora* | 152,731 | 84,402 | 16,969 | 25,680 | 130 | 83 | 36 | 8 | MK860971 |
| *Paeonia ludlowii* | 152,687 | 84,426 | 16,983 | 25,639 | 138 | 87 | 40 | 8 | NC_035623 |
| *Paeonia obovata* | 152,736 | 84,399 | 17,031 | 25,653 | 122 | 82 | 37 | 8 | JQ952561 |
| *Paeonia ostii* | 152,763 | 84,390 | 17,077 | 25,648 | 138 | 87 | 39 | 8 | MK701990 |
| *Paeonia qiui* | 152,578 | 84,242 | 17,044 | 25,646 | 131 | 84 | 37 | 8 | MT210544 |
| *Paeonia rockii* subsp. *rockii* | 152,840 | 84,494 | 17,052 | 25,647 | 123 | 83 | 37 | 8 |  |
| *Paeonia rockii* subsp. *taibaishanica* | 153,368 | 85,030 | 17,042 | 25,648 | 123 | 83 | 37 | 8 |  |
| *Paeonia suffruticosa* | 153,154 | 84,605 | 17,059 | 25,745 | 95 | 82 | 12 | 6 | JQ952559 |
| *Paeonia veitchii* | 152,682 | 84,398 | 16,978 | 25,653 | 132 | 84 | 37 | 8 | NC_032401 |
