# Supplementary material for: Niche divergence at the intraspecific level in an endemic rare peony (Paeonia rockii): A phylogenetic, climatic and environmental survey
Source: Front Plant Sci. 2022 Nov 1;13:978011. doi: 10.3389/fpls.2022.978011 (PMC9663928; doi:10.3389/fpls.2022.978011)
Supplement: Supplementary Figure 1 — Geographical distribution of Paeonia rockii. [file DataSheet_1.zip › supplementary materials/Table S4.docx]

**Table S4 The potential distribution area of the two *Paeonia* species in the 2050s and 2070s.**

| **Species** | **Model** | **Period** | **Area of each suitable region (× 10^4^ Km^2^)** | | | |
| --- | --- | --- | --- | --- | --- | --- |
|  |  |  | **Unsuitable region** | **Unchanged region** | **Expansion region** | **Contractionregion** |
| *Paeonia rockii* subsp.*rockii* | MIROC5 | Present **vs** RCP2.6-2050s | 878.81 | 28.66 | 19.58 | 21.83 |
|  |  | Present **vs** RCP4.5-2050s | 864.95 | 33.25 | 27.44 | 23.25 |
|  |  | Present **vs** RCP6.0-2050s | 897.36 | 0.73 | 1.03 | 49.76 |
|  |  | Present **vs** RCP2.6-2070s | 873.82 | 33.28 | 17.22 | 24.57 |
|  |  | Present **vs** RCP4.5-2070s | 866.10 | 20.35 | 30.15 | 32.29 |
|  |  | Present **vs** RCP6.0-2070s | 861.25 | 27.30 | 29.14 | 31.20 |
| *Paeonia rockii* subsp.*taibaishanica* | MIROC5 | Present **vs** RCP2.6-2050s | 899.83 | 13.51 | 16.57 | 18.98 |
|  |  | Present **vs** RCP4.5-2050s | 894.64 | 9.62 | 24.75 | 19.87 |
|  |  | Present **vs** RCP6.0-2050s | 879.76 | 25.43 | 20.64 | 24.06 |
|  |  | Present **vs** RCP2.6-2070s | 900.16 | 19.027 | 14.24 | 15.46 |
|  |  | Present **vs** RCP4.5-2070s | 894.86 | 10.94 | 16.55 | 26.54 |
|  |  | Present **vs** RCP6.0-2070s | 881.58 | 11.01 | 26.82 | 29.48 |
| *Paeonia rockii* subsp.*rockii* | BCC-CSM2-MR | Present **vs** ssp126-2050s | 890.80 | 25.55 | 7.58 | 24.94 |
|  |  | Present **vs** ssp245-2050s | 890.24 | 27.18 | 8.14 | 23.32 |
|  |  | Present **vs** ssp370-2050s | 888.11 | 27.84 | 10.27 | 22.66 |
|  |  | Present **vs** ssp126-2070s | 892.29 | 24.77 | 6.09 | 25.72 |
|  |  | Present **vs** ssp245-2070s | 885.76 | 27.88 | 12.62 | 22.61 |
|  |  | Present **vs** ssp370-2070s | 886.12 | 32.58 | 12.26 | 17.92 |
| *Paeonia rockii* subsp.*taibaishanica* | BCC-CSM2-MR | Present **vs** ssp126-2050s | 917.27 | 9.60 | 9.88 | 12.12 |
|  |  | Present **vs** ssp245-2050s | 910.58 | 13.43 | 9.06 | 15.81 |
|  |  | Present **vs** ssp370-2050s | 906.76 | 14.95 | 14.63 | 12.54 |
|  |  | Present **vs** ssp126-2070s | 916.87 | 9.57 | 9.92 | 12.52 |
|  |  | Present **vs** ssp245-2070s | 915.19 | 12.35 | 13.20 | 8.14 |
|  |  | Present **vs** ssp370-2070s | 914.24 | 14.30 | 9.19 | 11.15 |
