# Supplementary figures and images for: Niche divergence at the intraspecific level in an endemic rare peony (Paeonia rockii): A phylogenetic, climatic and environmental survey
Source: Front Plant Sci. 2022 Nov 1;13:978011. doi: 10.3389/fpls.2022.978011 (PMC9663928; doi:10.3389/fpls.2022.978011)

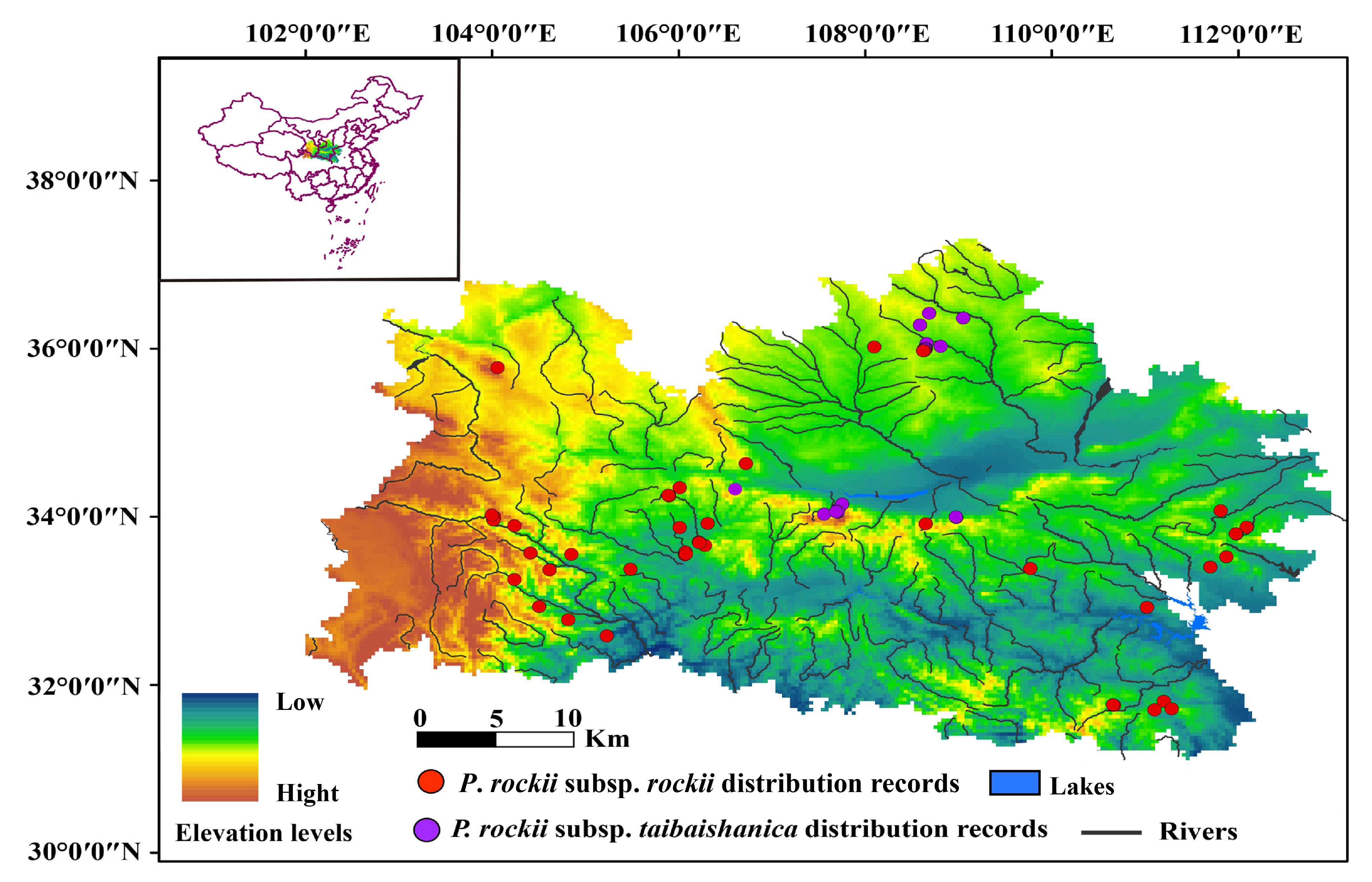

Supplement: Supplementary Figure 1 — Geographical distribution of Paeonia rockii. [file DataSheet_1.zip › supplementary materials/Figure S1.tif]

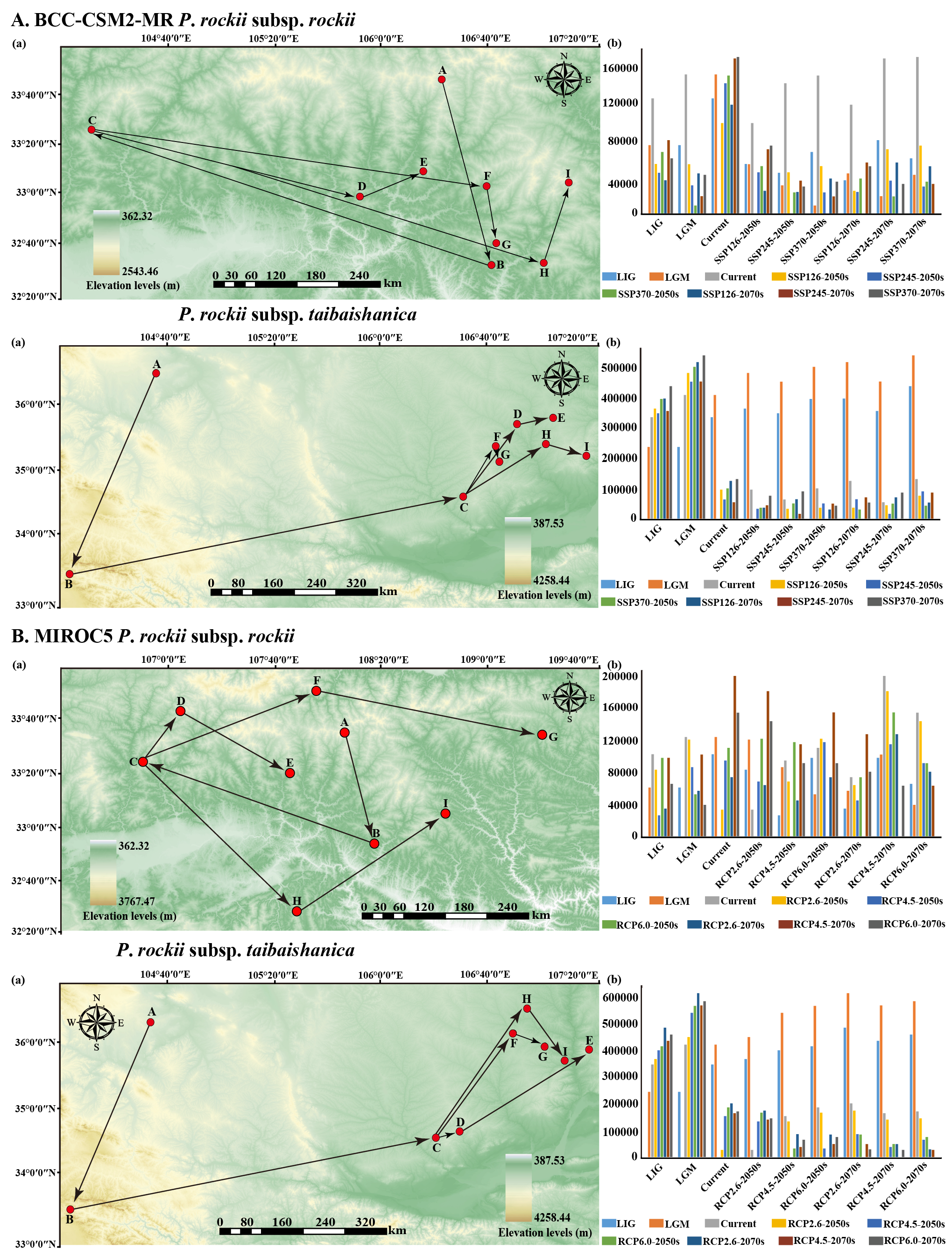

Supplement: Supplementary Figure 1 — Geographical distribution of Paeonia rockii. [file DataSheet_1.zip › supplementary materials/Figure S10.tif]

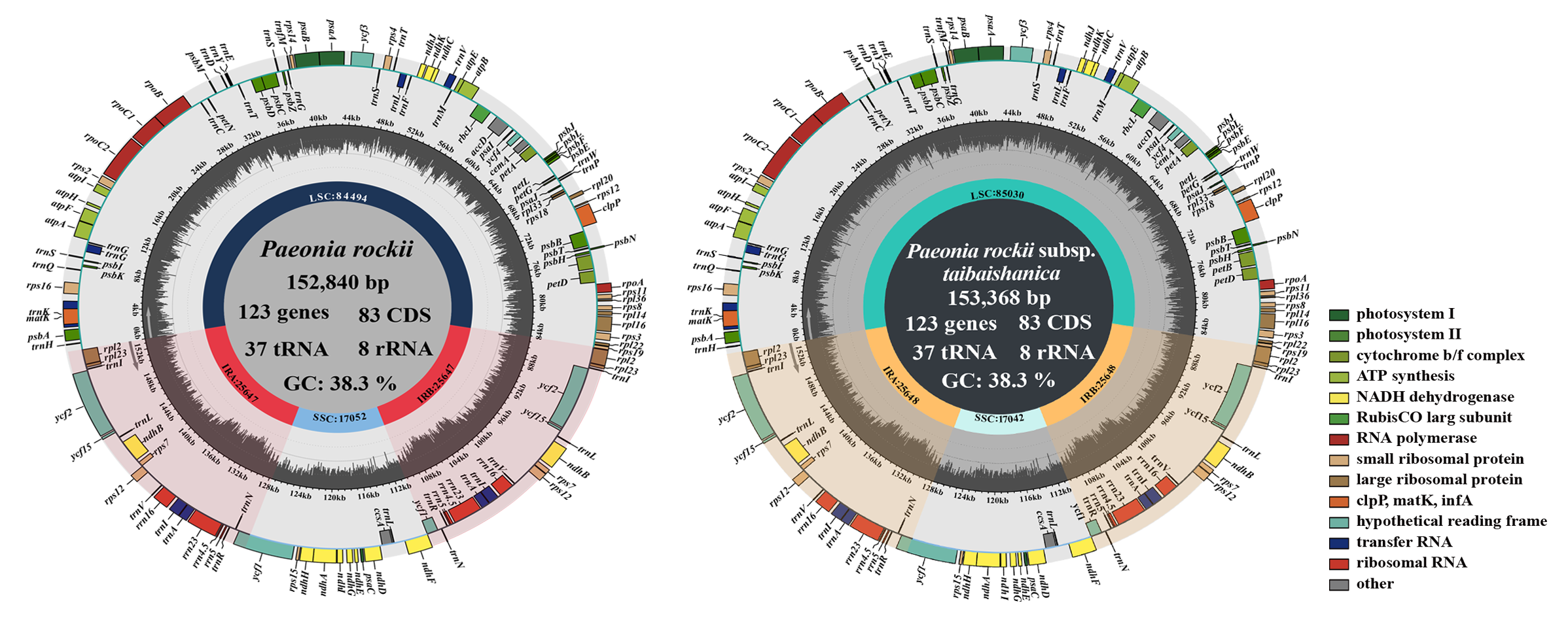

Supplement: Supplementary Figure 1 — Geographical distribution of Paeonia rockii. [file DataSheet_1.zip › supplementary materials/Figure S2.tif]

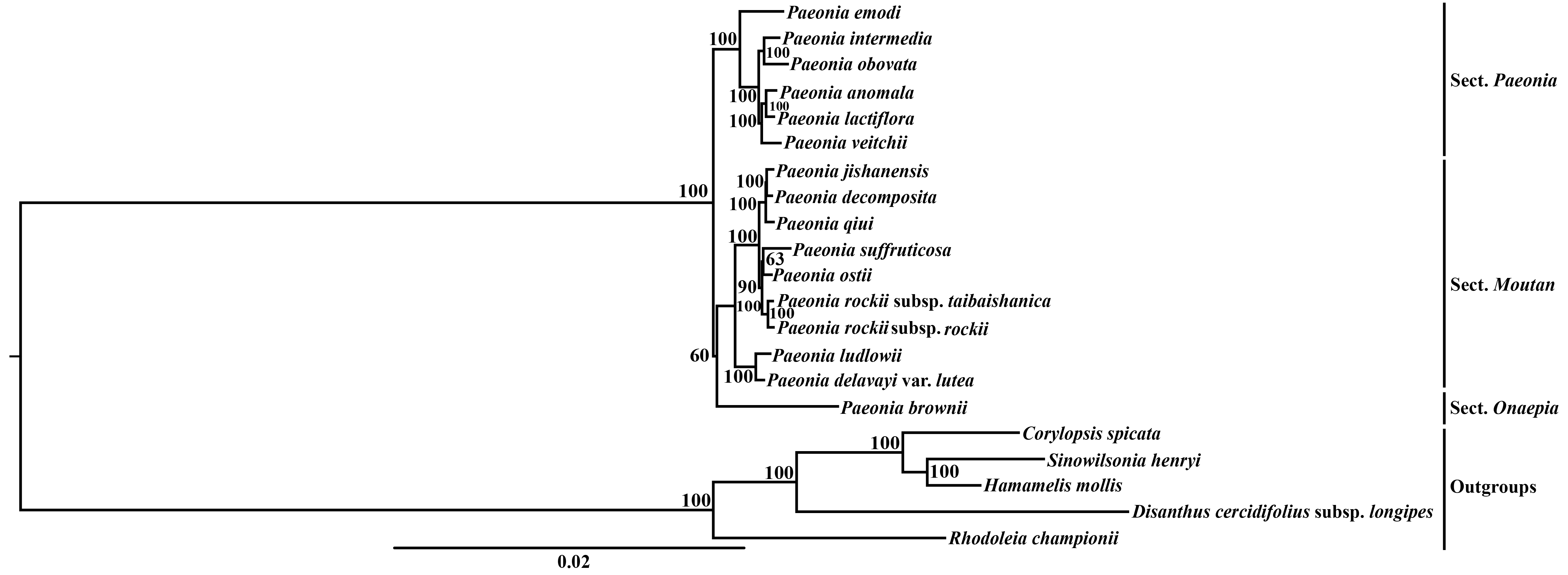

Supplement: Supplementary Figure 1 — Geographical distribution of Paeonia rockii. [file DataSheet_1.zip › supplementary materials/Figure S3.tif]

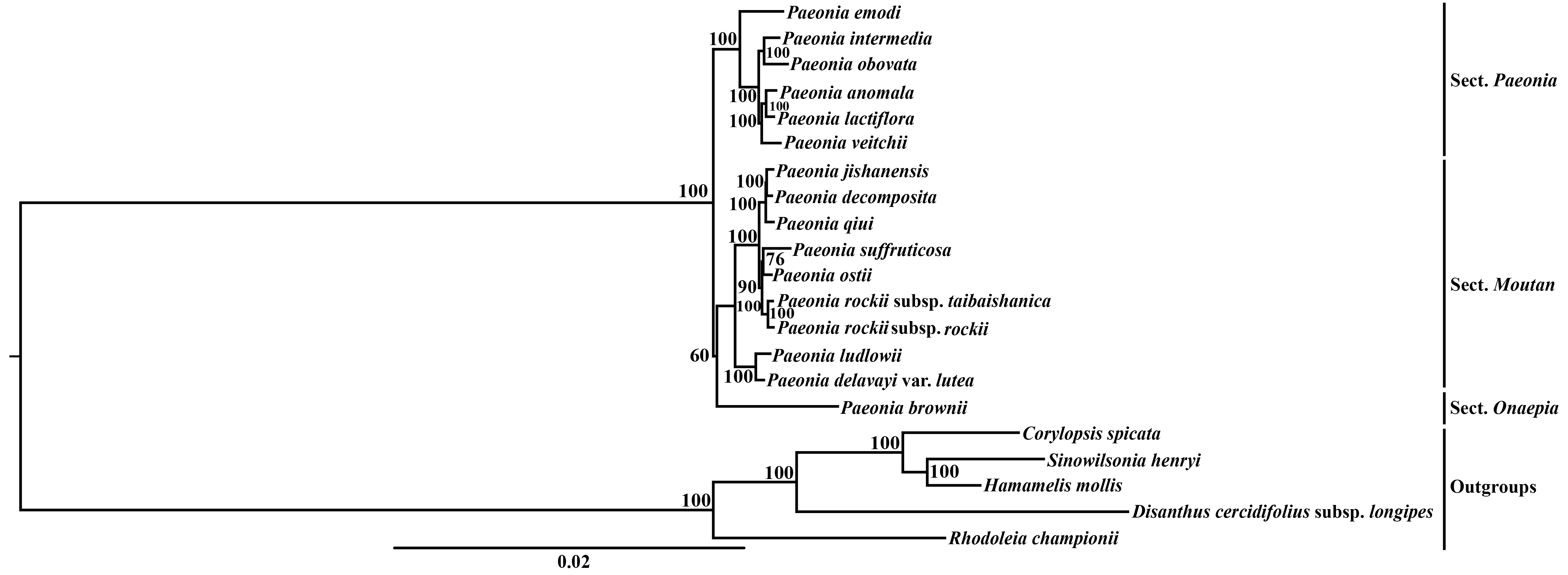

Supplement: Supplementary Figure 1 — Geographical distribution of Paeonia rockii. [file DataSheet_1.zip › supplementary materials/Figure S4.tif]

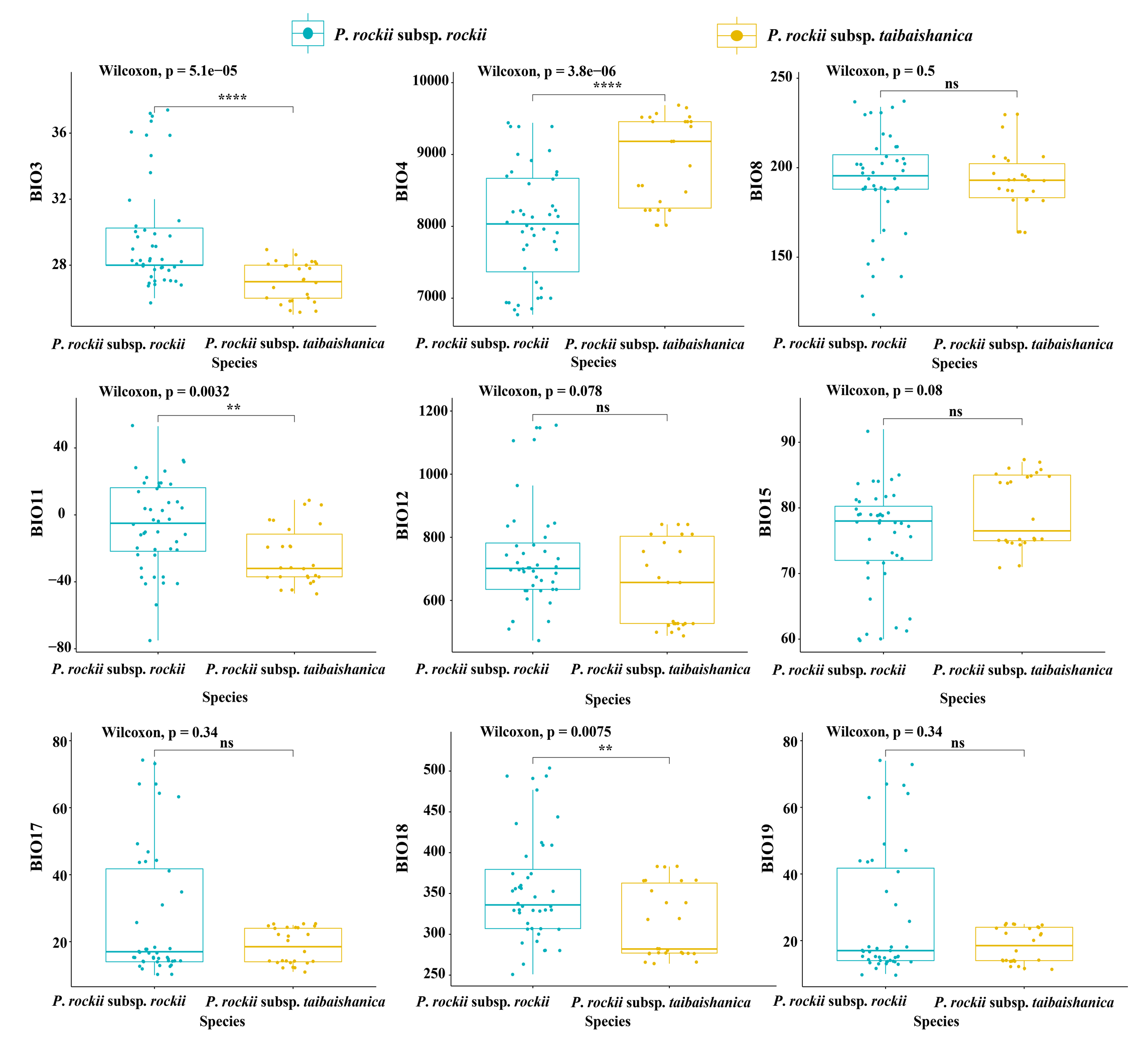

Supplement: Supplementary Figure 1 — Geographical distribution of Paeonia rockii. [file DataSheet_1.zip › supplementary materials/Figure S5.tif]

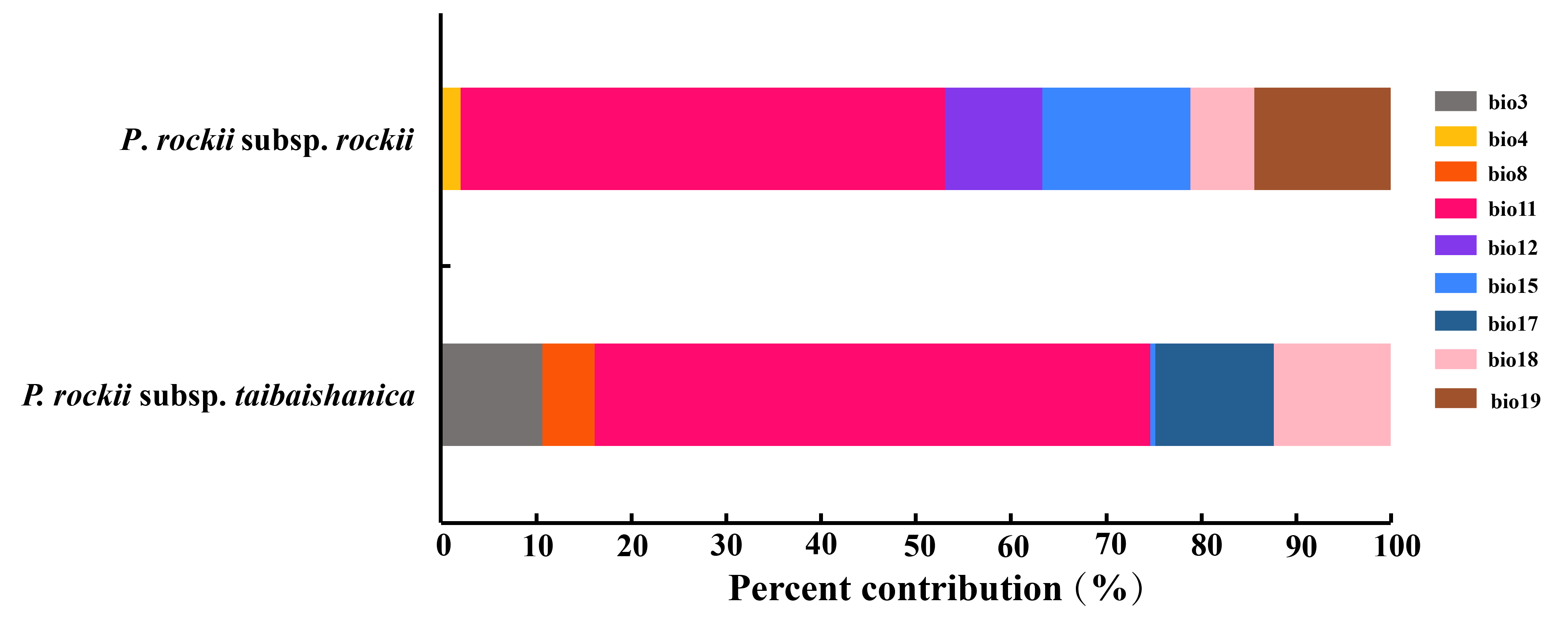

Supplement: Supplementary Figure 1 — Geographical distribution of Paeonia rockii. [file DataSheet_1.zip › supplementary materials/Figure S6.tif]

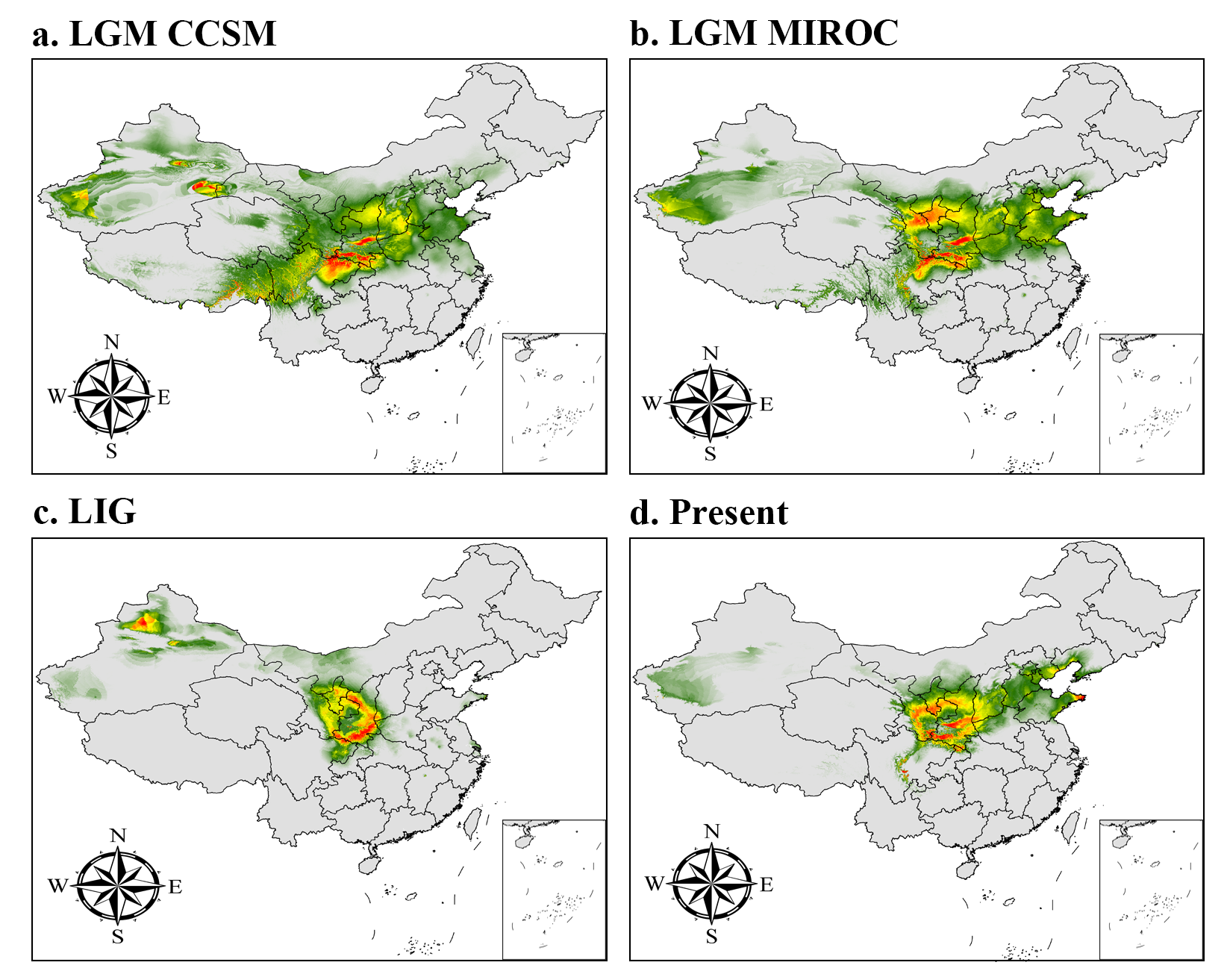

Supplement: Supplementary Figure 1 — Geographical distribution of Paeonia rockii. [file DataSheet_1.zip › supplementary materials/Figure S7.tif]

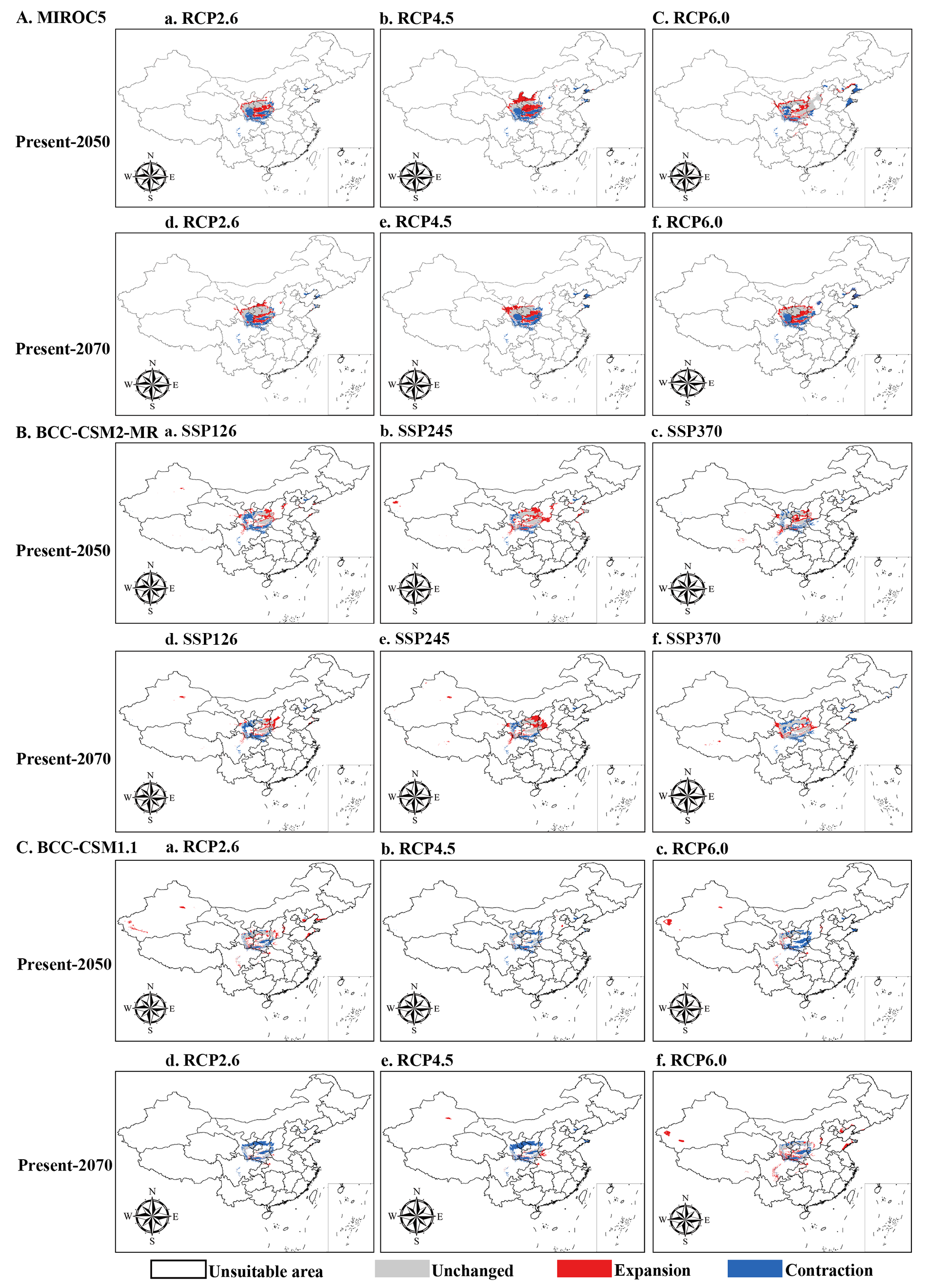

Supplement: Supplementary Figure 1 — Geographical distribution of Paeonia rockii. [file DataSheet_1.zip › supplementary materials/Figure S8.tif]

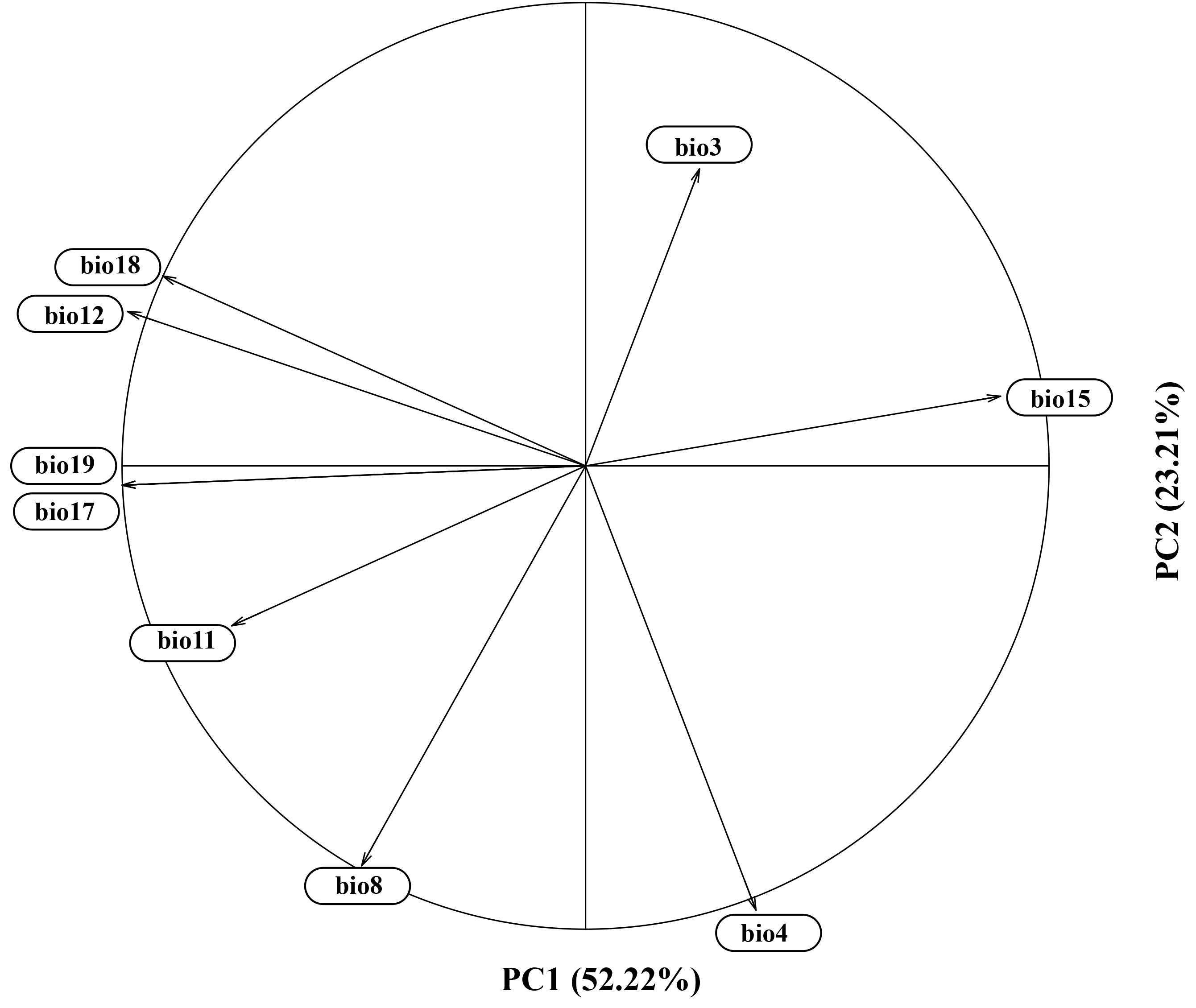

Supplement: Supplementary Figure 1 — Geographical distribution of Paeonia rockii. [file DataSheet_1.zip › supplementary materials/Figure S9.tif]
